# Identification of c‐Met on Tumor Cells as a Novel Receptor for B7‐H3 Entails Implications for Cancer Cell Stemness and Targeted Therapy

**DOI:** 10.1002/mco2.70332

**Published:** 2025-08-22

**Authors:** Lei Cao, Yunyun Xu, Yizhou Hu, Xue Huang, Fengqing Fu, Shenghua Zhan, Lili Huang, Yangyang Feng, Ylivinkka Irene, Huini Li, Varjosalo Markku, Keski‐Oja Jorma, Guangbo Zhang, Binfeng Lu, Jian Wang, Wanli Liu, Xueguang Zhang

**Affiliations:** ^1^ Jiangsu Institute of Clinical Immunology & Jiangsu Key Laboratory of Clinical Immunology The First Affiliated Hospital of Soochow University Suzhou China; ^2^ Institute of Pediatrics Children's Hospital of Soochow University Suzhou China; ^3^ Research Program Faculty of Medicine University of Helsinki and The Hospital District of Helsinki and Uusimaa Helsinki Finland; ^4^ Division of Molecular Neurobiology Department of Medical Biochemistry and Biophysics Karolinska Institute Stockholm Sweden; ^5^ State Key Laboratory of Radiation Medicine and Protection Soochow University Suzhou China; ^6^ MOE Key Laboratory of Protein Sciences Center for Life Sciences School of Life Sciences Beijing Key Lab for Immunological Research on Chronic Diseases Institute for Immunology Tsinghua University Beijing China; ^7^ Institute of Biotechnology and Helsinki Institute of Life Science University of Helsinki Helsinki Finland; ^8^ Department of Immunology School of Medicine University of Pittsburgh Pittsburgh Pennsylvania USA; ^9^ Soochow University‐Bright Scistar Antibody Joint Laboratory Suzhou China

**Keywords:** B7‐H3, cancer cell stemness, monoclonal antibody, molecular targeted therapy

## Abstract

The immune checkpoint molecule B7‐H3 is upregulated in many solid tumors, and B7‐H3‐targeted immunotherapies are in clinical trials. Recently, a growing body of research has highlighted the presence of tumor cell intrinsic while immune cell‐independent functions of B7‐H3 in tumorigenesis and cancer cell stemness. However, its receptors and mechanisms of action on cancer cells remain poorly understood. Here, we report that c‐Met, a canonical oncogenic receptor tyrosine kinase on cancer cells, is identified as a novel binding protein for B7‐H3. The binding between c‐Met and B7‐H3 directly activates the c‐Met/STAT3 signaling cascade, promoting cancer cell stemness in both colorectal cancer and glioblastoma‐derived tumor cells. More importantly, we evaluated the translational implications of this discovery by screening a high‐affinity antibody designed to selectively disrupt the interaction between B7‐H3 and c‐Met, demonstrating strong anti‐tumor activities, surpassing that of the B7‐H3‐specific antibody lacking the blocking capability. Combination therapy of this newly developed interaction blocking antibody with c‐Met inhibitor results in significantly improved therapeutic effects in inhibiting tumor growth. These findings shed light on previously undisclosed interaction of B7‐H3 to c‐Met on cancer cells, thereby indicating a new mechanism of cancer cell stemness and intervention pathway of molecular targeted therapy.

## Graphical abstract

B7‐H3 and c‐Met interaction is revealed to be crucial in maintaining cancer cell stemness primarily via c‐Met/STAT3 signaling pathway. B7‐H3 antibody blocking this newly identified interaction exhibits preclinical effects as a new and potent intervention for molecular targeted therapy.

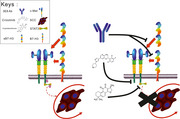

## 1 Introduction

The B7 family of cell surface proteins are typically co‐stimulatory molecules known to be involved in the immune checkpoint as exemplified by B7‐H1 (PD‐L1). However, unlike B7‐H1, the function of B7‐H3 has been notably intriguing [1]. Similar to B7‐H1, the clinical significance of B7‐H3 in tumor therapy has been well documented, as elevated expression of B7‐H3 in various cancers is correlated with poor prognosis [2, 3, 4]. Clinically, B7‐H3 exhibits low expression in normal tissues while high expression in tumor tissues, which established it as a tumor‐associated antigen and a pivotal therapeutic target in oncology, leading to the assessment of B7‐H3 therapeutic antibodies in preclinical and Phase I–II clinical trials [5, 6, 7, 8]. Furthermore, there are also recent studies exploring the potentials of B7‐H3 as a Chimeric Antigen Receptor T‐Cell Immunotherapy (CAR‐T) cell target in both hematological and solid tumors [9, 10, 11, 12].

Despite all these clinical progressions, in contrast to PD‐L1, there are also substantial investigations suggesting that B7‐H3 on tumor cells plays crucial tumor cell‐intrinsic functions in enhancing survival and proliferation signaling pathways, including PI3K/Akt, Jak/STAT, NF‐κB‐p65/MAPK‐p38, and Ras/Raf/MEK [13, 14]. Additionally, we and others have found that B7‐H3 can modulate tumor cell growth by regulating aerobic glycolysis, energy metabolism, cholesterol metabolism, and other pathways, highlighting its diversity in mediating tumor biology functions [14, 15, 16]. Since many of these studies are performed in the absence of CD8^+^ T cells and even other types of immune cells, it has been long believed that B7‐H3 may exhibit immune cell‐independent functions to intrinsically enhance cancer cell growth, drug resistance, epithelial‐mesenchymal transition, and maintenance of stemness across different cancer types.

This speculation is highly echoed by the fact that the receptor on immune cells for B7‐H3 is still not found yet despite the tremendous efforts from a lots of immunology labs, and the precise mechanism by which it mediates tumor immune evasion remains unclear, rendering the PD‐1 equivalent receptor on CD8^+^ T cells serving for B7‐H3 absence. On the basis of all these facts, there is an intriguing hypothesis that rather than on immune cells, there may be a receptor for B7‐H3 on cancer cells, which is of genuine novelty and importance for cancer cell proliferation and stemness through the binding of this potential receptor and B7‐H3.

We addressed this long‐standing question in this study by reporting that c‐Met, a canonical oncogenic receptor tyrosine kinase (RTK), is identified as a novel binding protein for B7‐H3. The binding between c‐Met and B7‐H3 was found to be crucial in maintaining the cancer cell stemness of both glioblastoma (GBM)‐ and colorectal cancer (CRC)‐derived tumor cells primarily via the activation of c‐Met/STAT3 signaling pathway. Co‐expression of both B7‐H3 and c‐Met in tumor cells also predicted the poorest survival in clinical samples. Monoclonal antibodies (mAbs) specifically blocking the interaction between B7‐H3 and c‐Met have been developed and demonstrated to exhibit highly efficient therapeutic potential against tumor growth in vivo. Combination therapy with c‐Met inhibitor and mAb results in better therapeutic effect in inhibiting tumor growth in multiple mouse xenograft models. These findings shed light on previously undisclosed receptor and mechanisms of action of B7‐H3 on cancer cells, thereby indicating a potential intervention pathway of molecular therapy targeting to cancer cell stemness.

## 2 Results

### 2.1 Expression and Function of B7‐H3 in CRC

**FIGURE 1:**
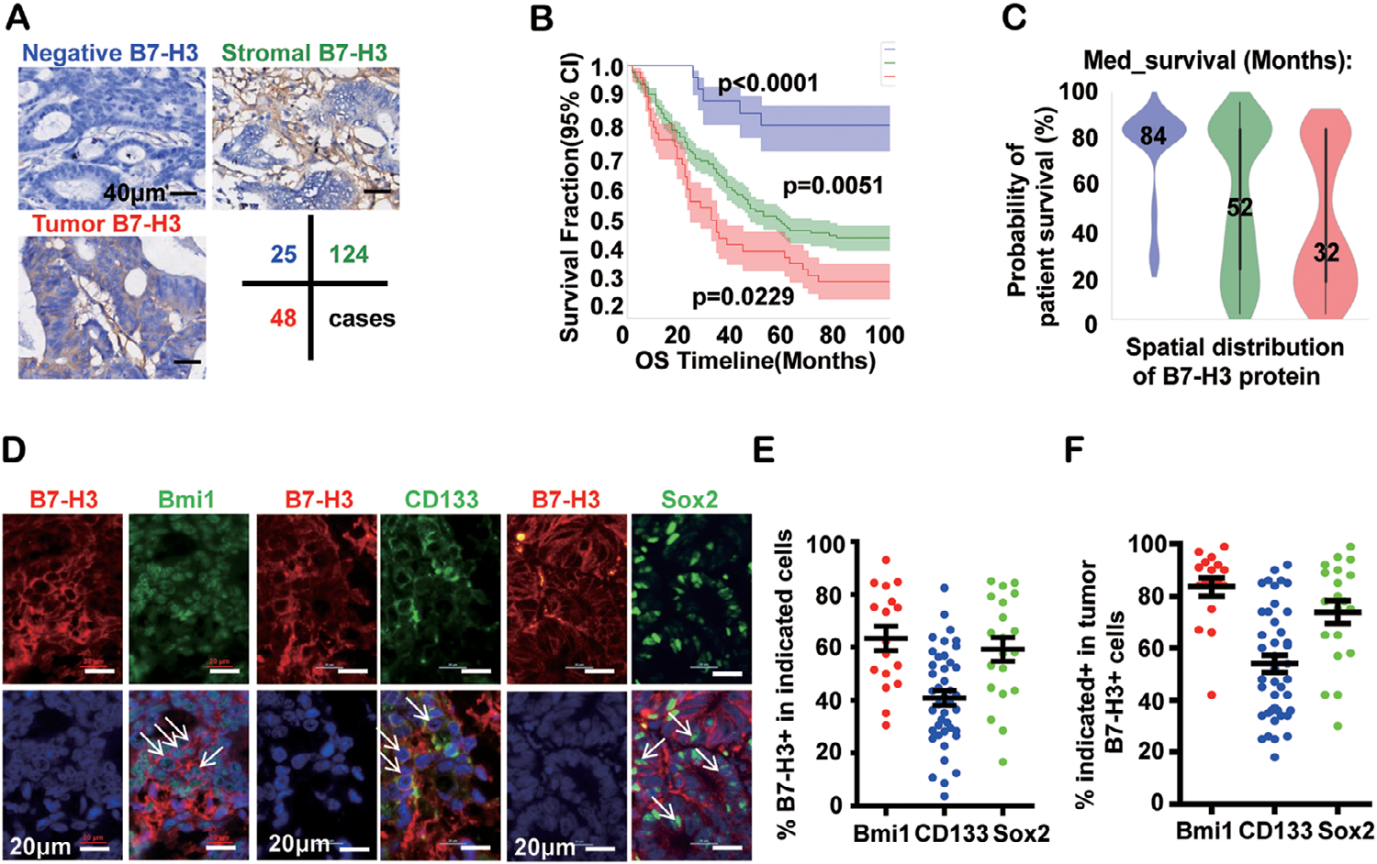
Tumor preferential expression of B7‐H3 predicts poor patient survival and labels cancer stem‐like cell in CRC patient tissue. (A) Representative immunohistochemical B7‐H3 staining of CRC‐resected human patient samples (total *n* = 197). Blue staining represents the nucleus, and brown staining represents B7‐H3 protein. B7‐H3 negative tissues rarely exhibit B7‐H3 staining (blue title *n* = 25). In stromal cell B7‐H3 expressing tissues, B7‐H3 is mainly located in the intercellular region (green title *n* = 124). In tumor cells expressing B7‐H3, the B7‐H3 protein predominantly accumulates in the membrane and cytoplasm (red title *n* = 48). Scale bars, 40 µm. (B) Kaplan–Meier survival curves of CRC subtypes as in (a). Negative, blue line; stromal cell B7‐H3, green line; tumor cell B7‐H3, red line. *Y*‐axis represents the survival fraction (95% CI), and *X*‐axis represents the timeline of patient survival. *p* value was calculated by log‐rank test and is shown in the figure. (C) Violin plots of patient survival in the different CRC subtypes: negative, blue plot; stromal cell, green plot; tumor cell, red plot. The width of each violin shape represents the ratio of patients with the indicated survival period in the subtype. (D) Representative immunofluorescence images for B7‐H3 and three cancer cell stemness markers Bmi1, CD133, and Sox2 in human patient CRC resected samples (*n* = 48). DAPI, blue; B7‐H3, red; Bmi1/Sox2/CD133, green, as indicated at the top of each image. Scale bars, 20 µm. (E) The percentage of B7‐H3 positive expression in Bmi1, CD133, or Sox2 positive cells, respectively. Error bars denote mean ± SEM. (F) The percentage of Bmi1, CD133, and Sox2 expression in B7‐H3 positive cells. Error bars denote mean ± SEM.

In order to explore the expression levels of B7‐H3 in patient tumor tissues, we studied the distribution of B7‐H3 protein in a cohort containing 197 CRC patients by immunohistochemistry. Histological observation revealed that B7‐H3 was expressed in both tumor cells and infiltrated stromal region cells in the tumor microenvironment (Figure 1A,B). Patients with B7‐H3 expression in tumor cells had the poorest prognosis, with a median survival expectation of only 32 months in comparison to 52 and 84 months in the stromal region B7‐H3 expression and negative B7‐H3 expression groups, respectively (Figure 1C, Table S1). Data from tissues and cell lines also indicated increased expression of B7‐H3 (Figure S1A–E). In addition, we found that three cancer cell stemness‐associated markers, Bmi1, CD133, and Sox2, were also expressed in the majority of B7‐H3 positive tumor cells in primary CRC samples from 48 patients (Figure 1D–F).

**FIGURE 2:**
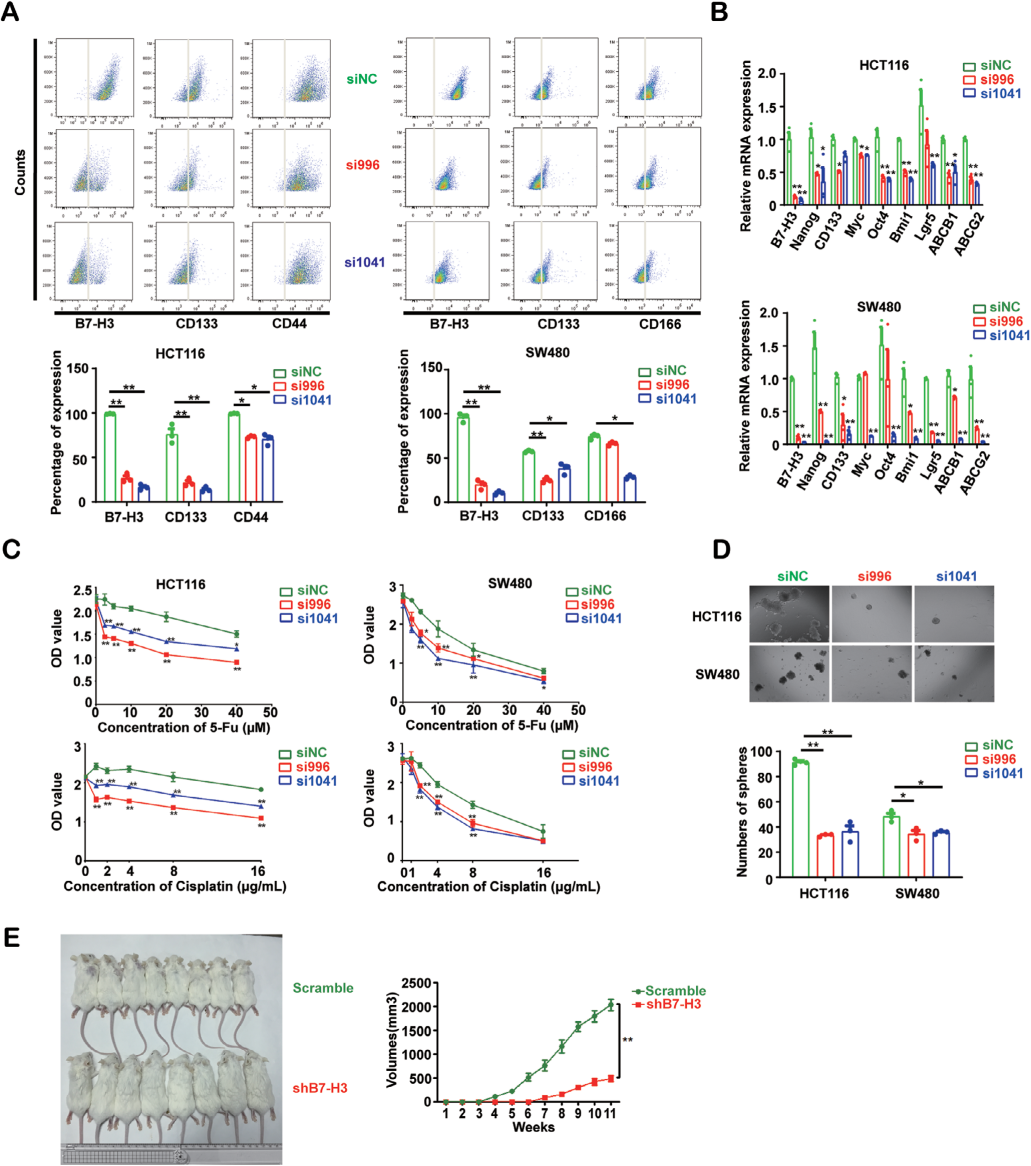
B7‐H3 knockdown reduced tumor stemness feature of tumor cells. (A) Flow cytometric analysis for B7‐H3, CD133, CD44, and CD166 expression in HCT116 and SW480 cells transduced without or with siRNA for B7‐H3. si996 (red) and si1041 (blue) are B7‐H3 siRNA, and green represents non‐targeting control siNC. (B) Representative real‐time PCR for tumor stemness related genes and B7‐H3 in HCT116 and SW480 cells transduced without or with siRNA for B7‐H3. Gene names are listed at *X*‐axis, and *Y*‐axis represents the relative expression. (C) The proliferation of HCT116 and SW480 cells transduced without or with siRNA for B7‐H3 after treatment with chemotherapeutic agents 5‐Fu or cisplatin. OD value represents the growth rate in each group, and drug concentrations are shown as *X*‐axis. The green circle, red square, and blue triangle indicate siNC, si996, and si1041, separately. (D) Representative images of sphere formation in HCT116 and SW480 cells transduced without or with B7‐H3 siRNA. The chart below represents the proportions of spheroid formation in each cell lines, si996 (red) and si1041 (blue) are B7‐H3 siRNA, and siNC (green) is non‐targeting control. Scale bars, 100 µm. (E) Subcutaneous growth of control and B7‐H3 knockdown HCT116 cells in SCID mice. Unpaired *t* test was used in (A), (B), and (D). One‐way ANOVA was used in (C) and (E). Data are representative of at least two independent experiments in (A)–(C). *n* = 8 mice per group in (E). Error bars denote SD in (A)–(D) and denote SEM in (E). **p < 0.01; *p value < 0.05.

To evaluate the effect of B7‐H3 on tumor stemness, we silenced B7‐H3 expression in HCT116 and SW480 cell lines by small interfering RNAs (siRNAs) and observed stemness‐associated markers downregulated expression both in protein and mRNA level (Figure 2A,B). Additionally, silencing B7‐H3 enhanced the chemotherapeutic effects of 5‐FU and cisplatin on the two cells (Figure 2C). Cells with reduced B7‐H3 expression also exhibited an impaired ability of spheroid formation (Figure 2D). Moreover, the HCT116 cells harboring B7‐H3 shRNA are rarely able to form tumor in vivo. The tumor formation of the human HCT116 cells emerged after 5 weeks, while there was no tumor in B7‐H3 gene silencing group (Figure 2E).

### 2.2 Direct Binding of B7‐H3 and c‐Met

**FIGURE 3:**
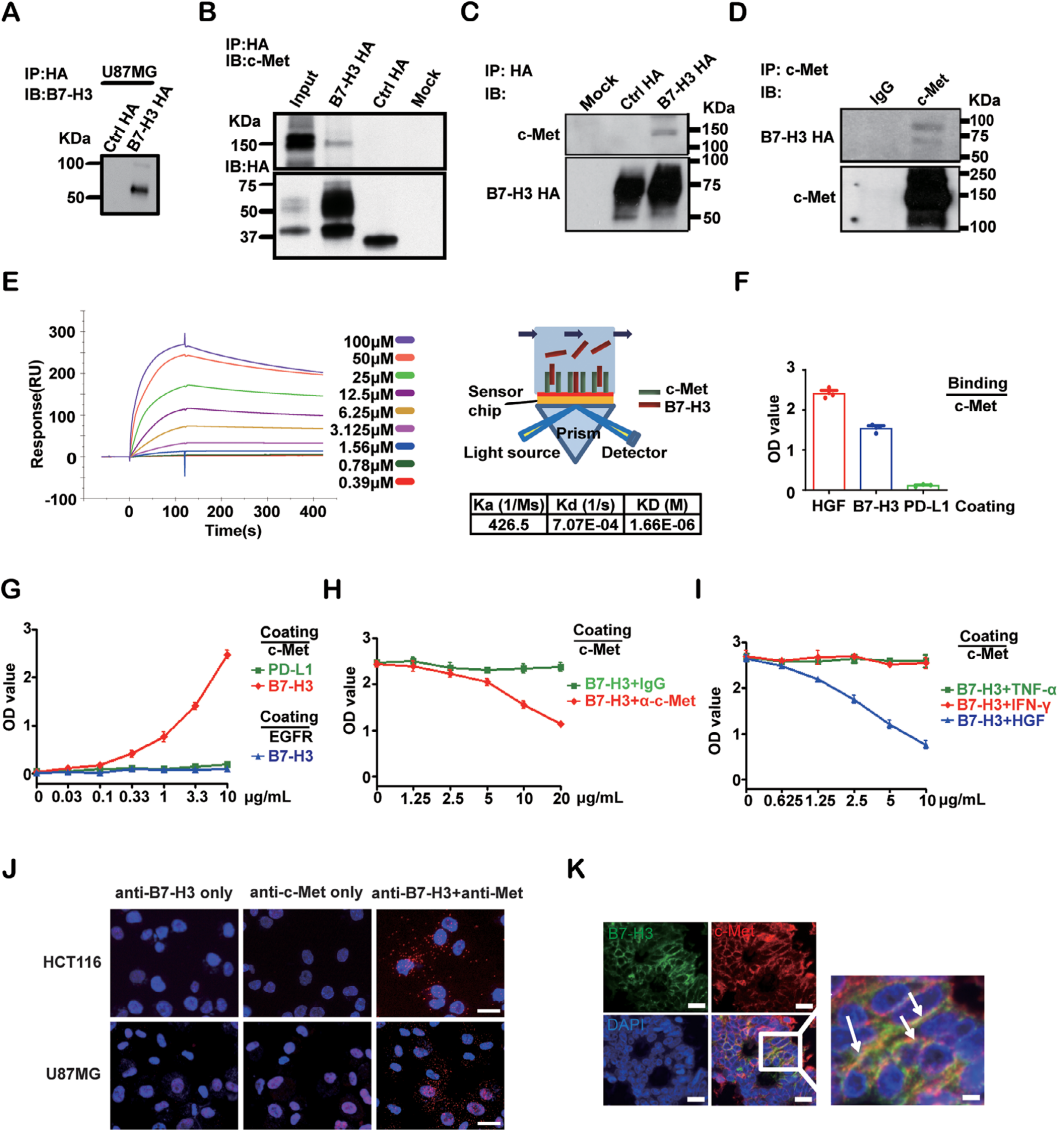
Direct and functional binding of B7‐H3 and c‐Met. (A and B) Immunoblotting validated the expression of HA‐tagged B7‐H3 in U87MG cells. The interaction between HA‐tagged B7‐H3 and endogenous c‐Met in U87MG cells was validated by immunoprecipitation against HA and immunoblotting against c‐Met. Empty vector was used as a mock control. HA‐tagged truncated netrin‐4 (30 kD) with a similar apparent molecular weight to c‐Met was used as an HA control. Cell lysates from U87MG overexpressing HA tagged B7‐H3 were used as a positive control. (C and D) The interaction between HA‐tagged B7‐H3 and c‐Met in SW480 cells was validated by co‐immunoprecipitation assays. Empty vector was used as a mock control. HA‐tagged netrin‐4 (75 kD) with a similar apparent molecular weight to B7‐H3 was used as an HA control. (E) The interaction between B7‐H3 and c‐Met was verified by SPR assays. Ka and kd were determined by kinetic fitting of the sensorgrams using SPR software, and the affinity constant (KD) was determined by calculating kd/ka. (F) ELISA validated the interaction between c‐Met and B7‐H3 (or HGF). HGF (1 µg/mL), B7‐H3 (5 µg/mL), or PD‐L1 (5 µg/mL, negative control) were incubated with the c‐Met pre‐coated (5 µg/mL) plates. Error bars denote mean ± SD from at least two independent experiments. (G) The interaction between B7‐H3 and c‐Met was confirmed by ELISA. Gradient B7‐H3 and PD‐L1 (negative control) at indicated concentrations were incubated with the c‐Met pre‐coated (10 µg/mL) plates. B7‐H3 at the indicated concentrations was incubated with PD‐1 pre‐coated (10 µg/mL) plates as a negative control. (H) The interfering potential of a c‐Met blocking antibody in B7‐H3 and c‐Met interaction. B7‐H3 (10 µg/mL) was pre‐incubated with either IgG control or the c‐Met blocking antibody α‐c‐Met at the indicated concentration, and then the mixtures were added to c‐Met pre‐coated (10 µg/mL) plates. (I) The interfering potential of HGF in B7‐H3 and c‐Met binding. B7‐H3 (10 µg/mL) was pre‐incubated with a gradient of TNF‐α, IFN‐γ, or HGF concentrations as indicated, and then the mixture was added to c‐Met pre‐coated (10 µg/mL) plates. TNF‐α and IFN‐γ were used as negative controls. In (G)–(I), error bars denote mean ± SD from at least two independent experiments. (J) Representative images of results obtained to investigate B7‐H3 and c‐Met interaction by Duolink in situ proximity ligation assay (PLA) assay in the indicated cells. The single B7‐H3 or c‐Met antibodies were used as controls. Scale bars, 20 µm. (K) Endogenous B7‐H3 (green) and c‐Met (red) immunofluorescence staining of CRC‐resected slides from patient samples. B7‐H3 co‐localized with c‐Met at the cell edge and in cytoplasm regions. Scale bars, CRC tissue 20 µm, magnified picture 5 µm.

To explore the interaction protein of B7‐H3 in cancer cell, we employ a TAP‐tag system overexpressing HA‐strep tagged B7‐H3, followed by mass spectrometry analyses (Figure 3A). A panel of high score interacting proteins of B7‐H3 were identified (Table S2) including a top candidate, c‐Met, which is a well‐characterized oncogene coding RTK with key functions in tumor metastasis and cancer cell stemness [17]. To validate this interaction initially acquired from mass spectrometry, we performed co‐immunoprecipitation, surface plasmon resonance (SPR), and enzyme linked immunosorbent assay (ELISA). In both U87MG and CRC cell line SW480 overexpressing HA‐strep tagged B7‐H3, immunoprecipitation with HA successfully pulled down c‐Met (Figure 3B,C). Conversely, HA‐tagged B7‐H3 protein was co‐immunoprecipitated with antibodies against c‐Met, but not an IgG control (Figure 3D). By using the purified recombinant protein of the extracellular domains of B7‐H3 (from Gly27 to Thr461) and c‐Met (from Met1 to Thr932), we validated their direct interaction by SPR, showing a tentative binding affinity of 1.66 µM (Figure 3E). Additionally, we performed ELISA experiments to show that B7‐H3 directly bound to c‐Met in a dose‐dependent manner, but not PD‐1, an irrelevant receptor (Figure 3F,G). Further competition ELISA experiments showed that the binding between B7‐H3 and c‐Met could be partially inhibited by both a c‐Met blocking antibody (Figure 3H) and the c‐Met canonical ligand HGF, but not irrelevant molecules TNF‐α or IFN‐γ (Figure 3I). Extensive intracellular interaction between B7‐H3 and c‐Met was observed as indicated by red punctate signals in HCT116 and U87MG cells via proximity ligation assay (PLA) assay (Figure 3J). No signals were observed when either of the primary antibodies was omitted. Moreover, immunofluorescence analysis in CRC tissues from patients revealed that B7‐H3 and c‐Met co‐localized on the plasma membrane and intracellular vesicle‐like structures (Figure 3K).

### 2.3 B7‐H3 Regulates Tumor Cell Stemness via c‐Met‐Stat3 Signal

**FIGURE 4:**
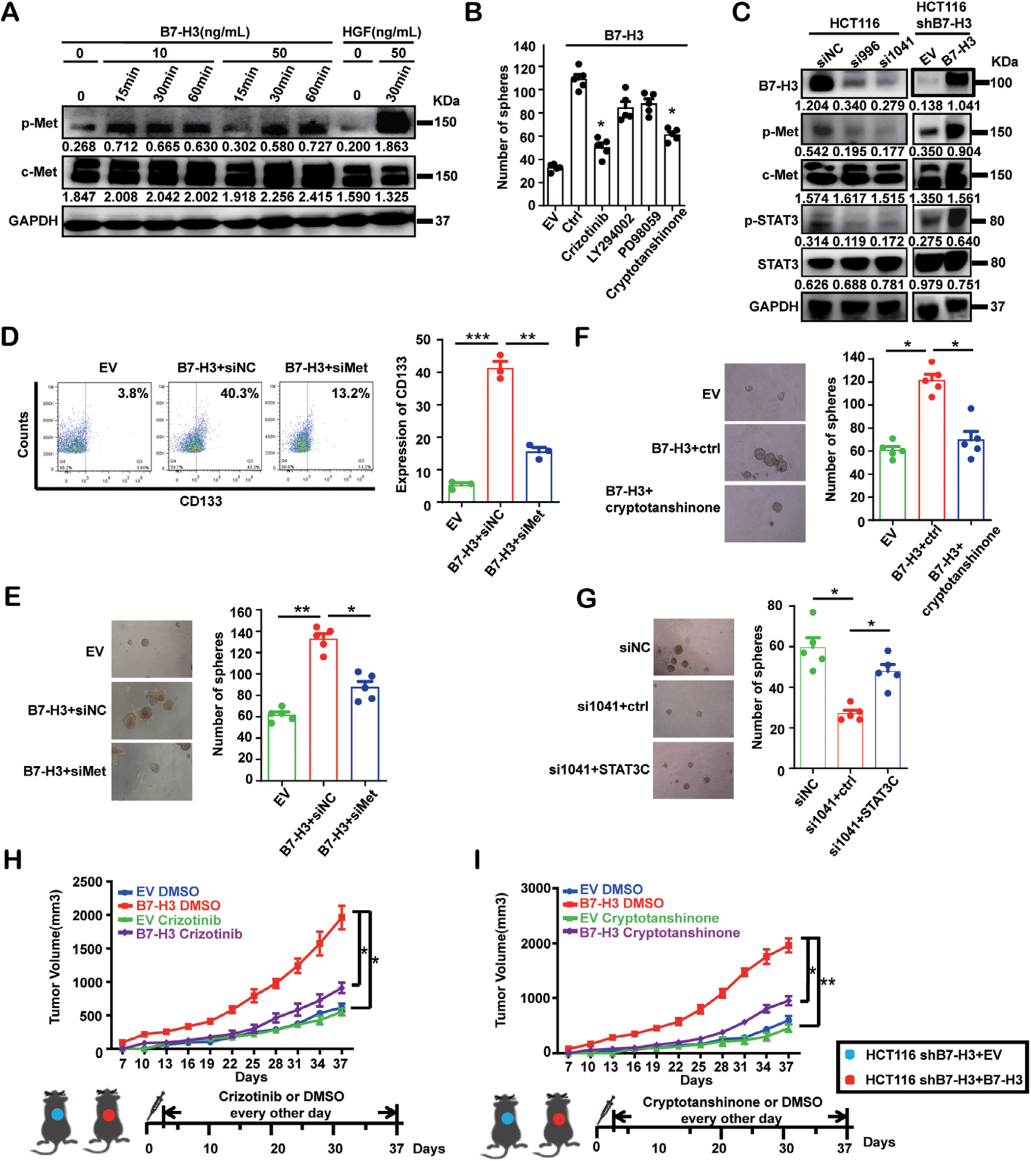
B7‐H3 regulates tumor cell stemness via c‐Met‐Stat3 signal. (A) Soluble recombinant B7‐H3 was used to stimulate the HCT116 cells at the indicated concentrations. The protein levels of c‐Met and the phosphorylation of c‐Met (Tyr1234/1235) in HCT116 cells were determined by immunoblotting. HGF was used as a positive control. Band intensities were quantified and indicated. (B) Quantification of spheroid numbers in B7‐H3 overexpressing HCT116 cells after treatment with inhibitors or controls. HCT116 cells were first silenced for B7‐H3 and then restored by lentivirus‐based overexpression (empty vector [EV] as a control). These cells were treated with a c‐Met inhibitor crizotinib, a PI3K inhibitor LY290042, a MAPK inhibitor PD98059, or a STAT3 inhibitor cryptotanshinone. DMSO was used as negative control. The bar chart presented the quantification results. Statistical analysis was performed using unpaired two‐tailed *t* test. Error bars denote mean ± SD from three independent experiments. (C) The phosphorylation of c‐Met and STAT3 is positively associated with the expression levels of B7‐H3. B7‐H3 was silenced by either si996 or si1041 in HCT116 cells, and siNC was used as non‐targeting control. B7‐H3 levels were restored by lentivirus‐based overexpression (EV as a control). The protein levels of B7‐H3, c‐Met, and STAT3, and the phosphorylation levels of c‐Met and STAT3 (Tyr705) in HCT116 cells were determined by immunoblotting. GAPDH was used as a loading control. Band intensities were quantified and indicated. (D) Flow cytometric analyses for CD133 expression in HCT116 cells overexpressing B7‐H3 (EV as a control) with or without siRNA‐based c‐Met knockdown. *Y*‐axis represents the intensity of fluorescence. (E) Quantification of the spheroid numbers in HCT116 cells overexpressing B7‐H3 (EV as a control) with or without siRNA‐based c‐Met knockdown. Bar chart represents the results of the quantification. (F) Quantification of the spheroid numbers in HCT116 cells overexpressing B7‐H3 (EV as a control) with or without treatment with the STAT3 inhibitor, cryptotanshinone. DMSO was used as a control. (G) Quantification of the spheroid numbers in HCT116 cells with B7‐H3 knocked down (si1041 group with siNC as a control). The reduction was rescued by the transfection of STAT3C, promoting STAT3 activation. In (D)–(G), unpaired two‐tailed *t* test was used for statistical analysis. ***p value < 0.001, **p value < 0.01, *p value < 0.05. Error bars denote mean ± SD from at least two independent experiments. (H and I) Subcutaneous growth of colon HCT116 tumors overexpressing B7‐H3. HCT116 cells were first silenced for B7‐H3 expression and then restored by lentivirus‐based overexpression (shB7‐H3+EV/B7‐H3). One‐week post inoculation, mice were sorted into four groups of equal average tumor burden and treatments were initiated with DMSO control (shB7‐H3+EV/B7‐H3+DMSO), c‐Met inhibitor crizotinib (shB7‐H3+EV/B7‐H3+crizotinib) (H), or STAT3 inhibitor cryptotanshinone (shB7‐H3+EV/B7‐H3+cryptotanshinone) (I) every other day for 32 days. Tumor sizes were evaluated every 3 days. In (H) and (I), statistical analysis was performed using one‐way ANOVA. **p value < 0.01; *p value < 0.05, *n* = 5 mice per group. Error bars denote mean ± SEM.

Next, we assessed the physiological function of this newly identified B7‐H3 and c‐Met interaction. The addition of recombinant B7‐H3 protein of its extracellular domain triggered the Tyr1234/1235 phosphorylation of endogenous c‐Met in HCT116 (a CRC cell line) cells, a feature of c‐Met receptor activation and Tyr705 phosphorylation of STAT3, a core c‐Met downstream signaling molecule (Figure 4A, Figure S2A) [17]. The exogenous expression of B7‐H3 also significantly enhanced the spheroid formation of HCT116 cells, a marker of CRC stemness feature [18], and this effect was significantly suppressed by either crizotinib, a c‐Met inhibitor, or cryptotanshinone, a STAT3 inhibitor (Figure 4B). In line with this observation, crizotinib suppressed the phosphorylation of both c‐Met and STAT3, which was triggered by the exogenous expression of B7‐H3 (Figure S2B). By complementing these inhibitor‐derived results, we found that silencing B7‐H3 in both HCT116 and U87MG cells attenuated the phosphorylation of c‐Met and STAT3, whereas overexpression of B7‐H3 enhanced the phosphorylation of c‐Met and STAT3 (Figure 4C, Figure S2C). Conversely, silencing c‐Met significantly reduced three features of CRC stemness [18, 19], the CD133 expression levels, the spheroid formation, and the drug resistance, upon B7‐H3 overexpression (Figure 4D,E, Figure S3A,B). Likewise, B7‐H3 overexpression‐induced spheroid formation was inhibited by cryptotanshinone (Figure 4F, Figure S3C), while the attenuated cancer cell stemness phenotypes observed upon silencing B7‐H3 were rescued by the transfection of STAT3C to promote STAT3 activation (Figure 4G, Figure S3D,E). Then, we examined whether these in vitro observations using all these different types of tumor cell lines could be recapitulated in vivo. Indeed, B7‐H3 overexpression readily promoted the growth of HCT116 cells in mouse xenograft model studies, the effect of which was significantly suppressed by both crizotinib and cryptotanshinone treatment (Figure 4H,I). In contrast, these suppression effects were not observed in HCT116 cells knocked down for B7‐H3 expression (Figure 4H,I), revealing B7‐H3 as the key molecule triggering CRC stemness. Thus, all these data collectively indicate that B7‐H3 potentiates cancer cell stemness through c‐Met/STAT3 signaling.

### 2.4 B7‐H3 Antibodies Blocking the B7‐H3 and c‐Met Interaction Have Anti‐Tumor Activity

**FIGURE 5:**
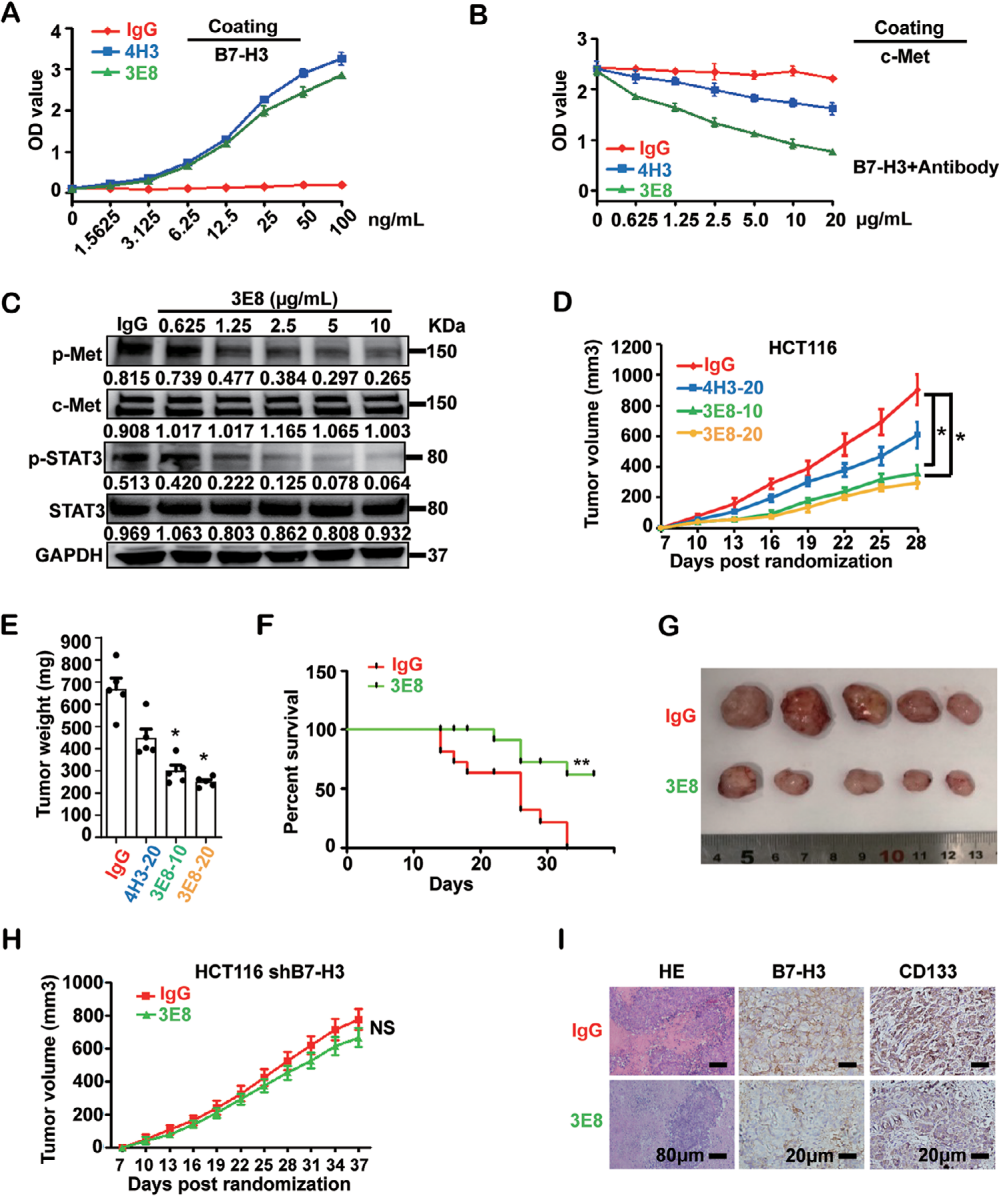
B7‐H3 antibodies blocking the B7‐H3 and c‐Met interaction have anti‐tumor activity. (A) ELISA validation of B7‐H3‐specific mAbs, 4H3 and 3E8. Control IgG (red), 4H3 (Blue), or 3E8 (green) was incubated with B7‐H3 pre‐coated (10 µg/mL) plates at indicated concentrations. (B) B7‐H3 (5 µg/mL) was pre‐incubated with B7‐H3‐specific mAbs 3E8, 4H3, or control IgG at the indicated concentration, and then the mixtures were added to c‐Met pre‐coated (5 µg/mL) plates. Binding was determined by ELISA. In (A) and (B), error bars denote mean ± SD from at least two independent experiments. (C) HCT116 cells were pre‐treated with the 3E8 mAb at the indicated concentrations in vitro, and c‐Met, p‐Met, STAT3, and p‐STAT3 levels were evaluated by immunoblotting. IgG antibody was used as a negative control, and GAPDH as a loading control. Band intensities were quantified and indicated. (D) Subcutaneous growth of colon HCT116 tumor cells in mice treated with 3E8 mAb (10 mg/kg, 20 mg/kg), 4H3 mAb (20 mg/kg), or IgG control. At the end point of 36 days, the tumor volumes in both the 3E8‐10 (10 mg/kg, green line) and 3E8‐20 groups (20 mg/kg, yellow line) were threefold smaller than in the IgG control group (red line). Statistical analysis was performed using one‐way ANOVA. *p value < 0.05, *n* = 5 mice per group. Error bars denote mean ± SEM. (E) The weights of the colon HCT116 tumors in mice treated with 3E8 mAb (10 mg/kg, 20 mg/kg), 4H3 mAb (20 mg/kg), or IgG control. Unpaired two‐tailed *t* test was used for statistical analysis. *p value < 0.05, *n* = 5 mice per group. Error bars denote mean ± SD. (F and G) Survival curves and representative tumor images of colon HCT116 tumor cell bearing mice treated with 3E8 mAb (10 mg/kg) or IgG control. In (f), statistical analysis was performed using log‐rank test. **p value < 0.01. *n* = 8 mice per group. (H) Subcutaneous growth of colon HCT116 shB7‐H3 tumor cells in mice treated with 3E8 mAb (10 mg/kg) or IgG control. The tumor volumes throughout 37 days were marked in both groups. (I) HE, B7‐H3, and CD133 immunohistochemical staining of xenograft tumor tissue from colon HCT116 tumor cell bearing mice treated with 3E8 mAb (10 mg/kg) or an IgG control. Scale bars, HE staining 80 µm, B7‐H3, CD133 staining 20 µm.

To evaluate the therapeutic potential of targeting B7‐H3 and c‐Met interaction, we developed a functional blocking antibody against the B7‐H3 and c‐Met recognition by generating a panel of B7‐H3 specific mAbs and evaluating their abilities to block the B7‐H3 and c‐Met recognition. Two mAbs, 3E8 and 4H3, blocked the B7‐H3 and c‐Met interaction in a dose‐dependent manner, with 3E8 showing greater blocking efficiency (Figure 5A,B, Figure S4A–D), although both mAbs showed high affinity and recognized a similar epitope on B7‐H3 (Figure S4E). In terms of function, 3E8 mAb inhibited the phosphorylation of c‐Met and STAT3 in a dose‐dependent manner upon B7‐H3 stimulation (Figure 5C). On the contrary, non‐blocking control antibody 4G4, even though can bind to B7‐H3, was unable to block c‐Met signal (Figure S5A). Treatment of xenograft nude mice with either 3E8 or 4H3 mAbs significantly suppressed HCT116 cells growth while treatment with 4G4 did not show such effects (Figure 5D,E, Figure S5B). Notably, 3E8 mAb treatment substantially improved survival of nude mice in HCT116 cells xenograft model (Figure 5F), with 60% surviving by the experimental endpoint of 37 days, whereas the control mice had to been executed on Day 34 because of the overburden of tumor volume. Tumors treated with 3E8 mAb were up to three times smaller than those treated with the IgG control at the experimental end point (4 weeks, Figure 5G), the effects of which were not obvious on tumor cells knocked down for B7‐H3 expression (Figure 5H). Tissue sections of tumors treated with the 3E8 mAb exhibited decreased staining of both B7‐H3 and CD133 compared with the IgG control group (Figure 5I), further supporting the reduced tumorigenecity upon blocking antibody treatment. In addition to CRC, 3E8 antibody also has a significant inhibitory effect on the growth of U87MG and gastric cancer cell NCI‐N87 (Figure S5C,D).

### 2.5 Computational Simulation Analysis of the B7‐H3‐c‐Met Interaction Blocking Antibody 3E8

**FIGURE 6:**
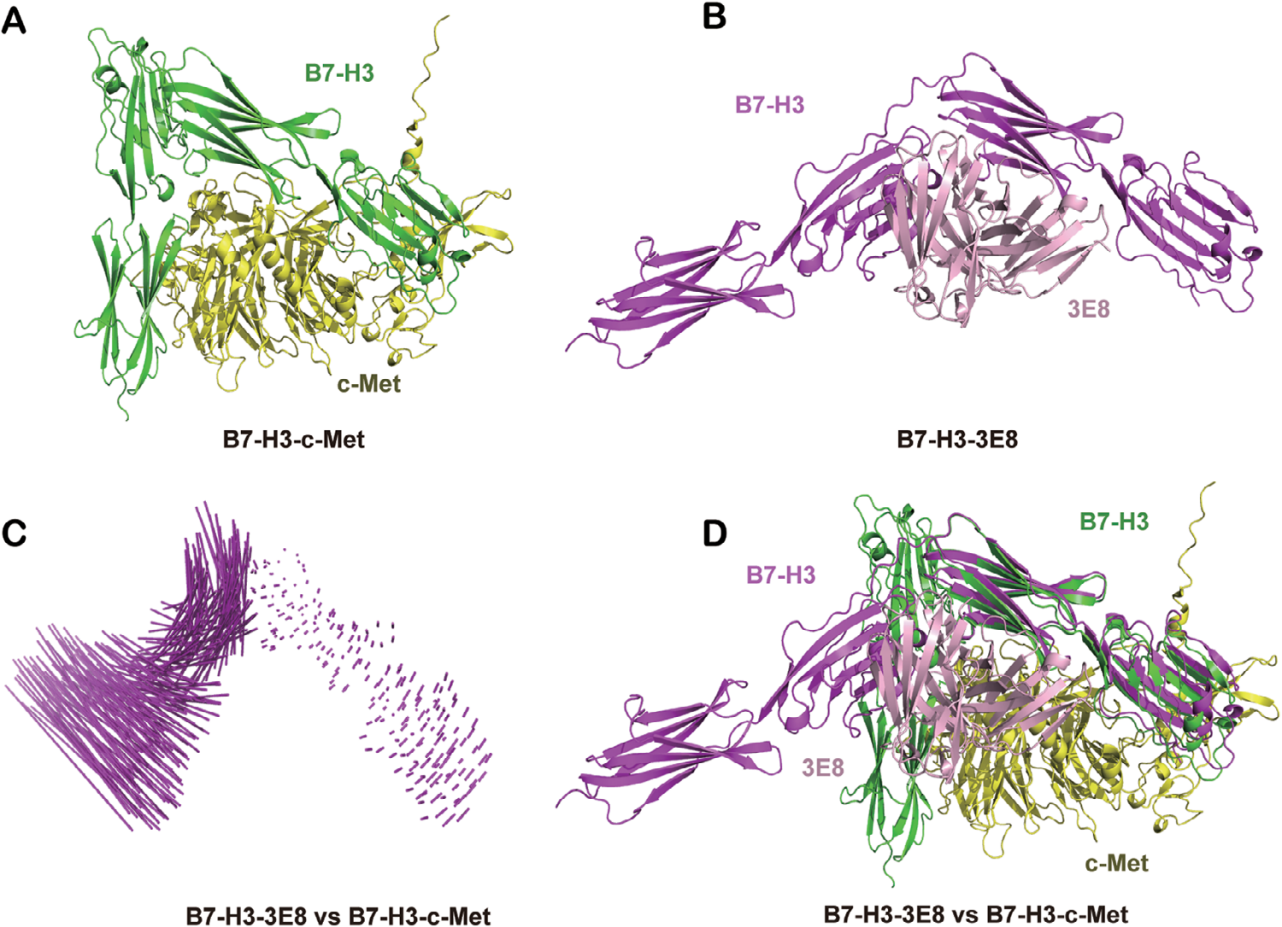
Computational simulation analysis of the B7‐H3 antibody 3E8 blocking the B7‐H3‐c‐Met interaction. (A) The complex structure of B7‐H3 with c‐Met (B7‐H3‐c‐Met) predicted using AlphaFold 3 in ribbon representation. (B) The complex structure of B7‐H3 with antibody 3E8 (B7‐H3‐3E8) in ribbon representation. (C) Superposition of B7‐H3 with c‐Met complex and B7‐H3 with antibody 3E8 complex showing the conformational change upon B7‐H3. (D) Superposition of B7‐H3‐c‐Met complex and B7‐H3‐3E8 complex.

We first used computational method to simulate the structure of the B7‐H3‐c‐Met complex by using AlphaFold 3, which revealed that c‐Met binds within the cleft of B7‐H3 (Figure 6A). To investigate how B7‐H3 interacts with the 3E8 antibody, we further employed AlphaFold 3 to predict the structure of the B7‐H3‐3E8 complex (Figure 6B). A comparative analysis between the B7‐H3‐c‐Met and B7‐H3‐3E8 complexes revealed a significant conformational change in B7‐H3 upon binding with 3E8 (Figure 6C). This structural modification induced by 3E8 may prevent B7‐H3 from interacting with c‐Met (Figure 6D). Comparative structural analysis of the B7‐H3‐c‐Met and B7‐H3‐3E8 complexes revealed distinct conformational states of B7‐H3. The 3E8‐induced structural alteration induces significant conformational rearrangements in B7‐H3 that sterically hinder its interaction interface with c‐Met. Through interface mapping analysis, we identified key amino acid residues within 4 Å of the binding interface in both complexes (Figure S6A,B). Notably, the 3E8‐bound conformation of B7‐H3 exhibits substantial topological divergence from its c‐Met‐binding state, particularly in regions critical for receptor engagement.

### 2.6 Patients With B7‐H3 and c‐Met Co‐expression Had the Worst Prognosis

**FIGURE 7:**
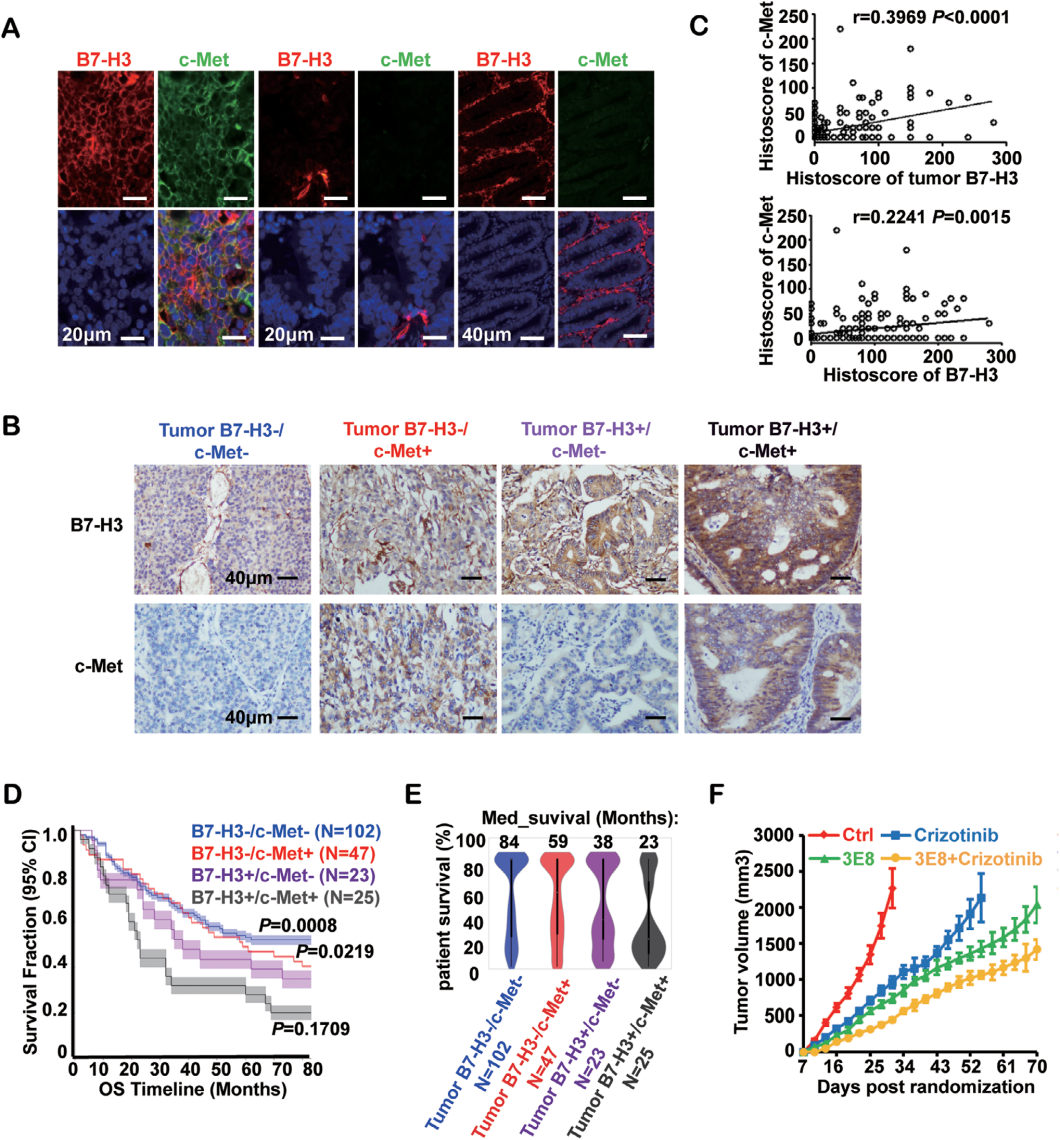
Patients with B7‐H3 and c‐Met co‐expression had the worst prognosis. (A) Representative immunofluorescence images for B7‐H3 (red) and c‐Met (green) staining in human patient CRC‐resected samples (*n* = 24). Three categories of expression pattern of these two molecules in CRC tissues are presented as follows: left panel, dual B7‐H3 and c‐Met co‐expression in tumor cells; middle panel, neither B7‐H3 nor c‐Met expression in tumor cells; right panel, preference of B7‐H3 expression alone in stromal regions. Scale bars, 20 µm or 40 µm as indicated. (B) According to the B7‐H3 location and c‐Met expression, 197 CRC patients were divided into four groups. Shown were representative images of B7‐H3 and c‐Met immunohistochemical staining of these four groups. B7‐H3 expression was located in both tumor cell and stromal region, whereas c‐Met expression was located in tumor cell. Scale bars, 40 µm. (C) The statistical correlation between B7‐H3 expression and c‐Met at protein levels in tumor cells (Up) or overall region (Down) are shown. Pearson's correlation coefficient test was used for correlation analysis. (D) 197 CRC patients were divided into four groups according to the location of B7‐H3 in the tissue section and its co‐expression with c‐Met as represented in (g), as shown in the figure key. Survival of these four groups was evaluated by Kaplan–Meier analysis. The dotted lines represent the significance of the difference between the two indicated groups, and *p* values were calculated using the log‐rank test. (E) Violin plot represented the distribution of patient survival in the four groups as detailed in (b); box plot in each violin shape describes the 25% percentile rank of patients at this probability of survival time. (F) Subcutaneous growth of colon HCT116 tumor cells treated with 3E8 mAb (10 mg/kg) alone, crizotinib alone, 3E8 mAb combined crizotinib, or IgG control. *n* = 5 mice per group. Error bars denote mean ± SEM.

To investigate the clinical relevance of the B7‐H3/c‐Met/STAT3 signaling axis in patient survival, we first retrieved both CRC and GBM patient data from the TCGA dataset since both CRC and GBM tumor cell lines have been examined in the in vitro biochemical studies in this report. We found that high co‐expression of B7‐H3/c‐Met, B7‐H3/STAT3, and B7‐H3/c‐Met/STAT3 predicted poor survival in both CRC (Figure S7A) and GBM patients (Figure S7B). More importantly, we performed immunostaining to examine the co‐expression of B7‐H3 and c‐Met in 197 CRC specimens from the cohort enrolled in this study (Figure 7A). A positive correlation was observed between the B7‐H3 and c‐Met expression levels (Figure 7B,C). Remarkably, further investigation to these 197 CRC patient data revealed that the co‐expression of both B7‐H3 and c‐Met in tumor cells predicted the shortest metastasis‐free survival in CRC patients (Figure 7D,E, Table S3).

Lastly, we evaluated the outcome of a combination therapy of the c‐Met inhibitor crizotinib and the 3E8 mAb in mouse xenograft model. Although either crizotinib or 3E8 mAb alone was able to inhibit tumor growth, the combination of these two resulted in the best therapeutic effect (Figure 7F). Together, these results reveal an unexpected crosstalk between immune‐checkpoints/RTKs in tumor cells. In a broader perspective, these findings demonstrate the potential implications of targeted cancer therapies via functional blocking of the B7‐H3 and c‐Met interaction.

## 3 Discussion

Our study revealed that B7‐H3 directly interacts with c‐Met, triggering c‐Met/STAT3 phosphorylation and enhancing cancer cell stemness in vitro and in vivo. B7‐H3, as a co‐stimulatory molecule, its function, and mode of action are not entirely consistent with PD‐L1. Although literature suggests that B7‐H3 can bind with TLT‐2 to promote T‐cell activation, the receptor for B7‐H3 on T cells has not yet been recognized [20]. Besides regulating T‐cell activation by binding to an unknown receptor on T cells, B7‐H3 can also exert biological functions by binding to proteins like IL20RA [21] and MVP [22]. In this report, we show that canonical c‐Met ligand, HGF, could partially compete with B7‐H3 for c‐Met, probably due to an overlapping binding interface between HGF and B7‐H3 in c‐Met, as in the case of the InlB and c‐Met interaction [23]. We believe that this newly identified c‐Met ligand, B7‐H3, is of potential importance as HGF transcripts expression in cancer tissues are lower than that of the corresponding normal tissues, and HGF protein levels are not significantly correlated with patient survival (data from GEPIA). Based on all these data, we speculate that HGF and c‐Met complex may require additional signaling for tumorigenesis. It is worth noting that in comparison to HGF, this report shows that B7‐H3 triggers c‐Met activation in a pattern that is relatively weaker but more sustainable, suggesting that B7‐H3 and HGF may initiate distinct dynamics of c‐Met degradation upon ligand binding. In line with this speculation, we also discover that B7‐H3 and c‐Met exhibit stable co‐distribution within the intracellular vesicles. The illumination of the binding interface between B7‐H3 and c‐Met in 3D crystal structure will further elucidate this point. Furthermore, *cis* versus *trans* binding experiments [24] may be able to better explain the similarities and differences between the newly identified binding of B7‐H3 & c‐Met and the classic binding of HGF & c‐Met. We also consider it possible that B7‐H3 could, like CD40, recruit other membrane microdomains or adhesion molecules to form membrane raft complex after combining with c‐Met and to initiate tyrosine phosphorylation of intracellular substrates in tumor cells [25]. Although our results support STAT3 as the primary signaling adaptor of B7‐H3/c‐Met for tumorigenicity, other functions of B7‐H3 mediated by alternative c‐Met downstream signal pathways shall not be excluded [26]. The launch of all these abovementioned potentials will further reveal the binding pattern, signaling mechanism and tumorigenic function of B7‐H3 and c‐Met interaction.

Our data show that blocking the B7‐H3 and c‐Met interaction could significantly suppress tumor growth. First, we provided anti‐tumor evidence of crizotinib in B7‐H3 positive tumor cells. Clinical trials have shown that crizotinib has a significant antitumor activity in Met amplified NSCLC and GEC, in which HGF/c‐Met binding is dispensable [27, 28, 29]. However, there is no reliable biomarker for crizotinib response. Aberrantly elevated expression of B7‐H3 has been described in various human malignancies and rarely in normal healthy tissues [26]. Thus, local expression of B7‐H3 in tumor cells may be a promising marker for crizotinib response in different cancer types. B7‐H3 has been used as a promising molecular target for various cancer treatment, such as CAR‐T and antibody‐dependent cell‐mediated cytotoxicity [30], indicating the great cancer therapeutic potential of B7‐H3 functional blocking antibody with specific protein–protein interaction target. By developing the functional blocking mAbs against the B7‐H3 and c‐Met interaction and analyzing their therapeutic effect, our work may provide a new and promising candidate for B7‐H3 based cancer therapy. Combining B7‐H3 blocking mAbs and crizotinib appeared to exhibit synergistic therapeutic effect in vivo, suggesting that, as a combinational treatment approach, blocking B7‐H3 and c‐Met signaling module may enhance the therapeutic efficiency in cancer treatment.

The results of the computational simulation experiments revealed that the structural modification induced by 3E8 binding prevents the interaction between B7‐H3 and c‐Met. Specifically, 3E8 binding triggers a structural rearrangement in B7‐H3, altering its binding site and disrupting the cleft where c‐Met would typically bind. This suggests that the interaction of B7‐H3 with 3E8 induces a conformational change that blocks the c‐Met binding site, thereby inhibiting B7‐H3's ability to engage with c‐Met. Constructing mutants at these cleft sites could theoretically impact the interaction between B7‐H3 and c‐Met.

To address concerns in applications, we assert that the uniqueness of our 3E8 mAb remains preserved. Our empirical validation, conducted on immunodeficient murine models, attests to the therapeutic efficacy of this mAb in mitigating both tumorigenesis and cancer cell stemness. Significantly, our observations underscore the distinctive nature of B7‐H3, independent of canonical tumor immune therapy, setting it apart from conventional checkpoint blockade mAb targeting alternate B7 family members such as PD‐1. In response to problems regarding the utilization of immunodeficient mice in our in vivo experiments, we wish to underscore the inherent disparities between murine and human B7‐H3. Specifically, murine B7‐H3 predominantly exists in a 2Ig‐formed configuration, whereas its human counterpart assumes a 4Ig‐B7‐H3 conformation. Moreover, the human B7‐H3‐specific antibody, 3E8, exhibits an inability to recognize murine B7‐H3. Consequently, the evaluation of the 3E8 antibody's impact on either WT or genetically modified murine models remains methodologically unfeasible, despite the extensive murine tools at our disposal. While we acknowledge the importance of investigating the roles of canonical immune cells, particularly T cells, in the detailed therapeutic mechanisms of the identified interaction and blocking antibody (3E8), we maintain that our primary emphasis on the unique interaction between human B7‐H3 and c‐Met is pivotal to the narrative we present.

While our study provides strong evidence supporting B7‐H3 as a novel binding partner for c‐Met and its role in promoting cancer cell stemness, several limitations should be acknowledged. First, although we demonstrated the direct interaction between B7‐H3 and c‐Met using multiple in vitro assays, further structural characterization, such as high‐resolution crystallographic or cryo‐EM studies, is needed to fully define the binding interface and conformational changes. Second, our in vivo studies were conducted in immunodeficient murine models, which, while suitable for assessing tumor‐intrinsic effects, do not fully recapitulate the influence of an intact immune system. Future investigations using immunocompetent or humanized models would be valuable in assessing the potential immunomodulatory roles of the B7‐H3/c‐Met axis. Third, although we observed strong therapeutic effects of our B7‐H3 blocking antibody in combination with crizotinib, the clinical relevance of this approach requires further validation in patient‐derived xenografts and clinical samples. Addressing these limitations in future studies will help to refine our understanding of B7‐H3/c‐Met interaction and its therapeutic implications.

In this study, we identify c‐Met as a novel interactor for B7‐H3, revealing its role in promoting cancer cell stemness via the c‐Met/STAT3 signaling axis. Our findings provide new insights into the tumor‐intrinsic function of B7‐H3 beyond immune regulation. The development of functional blocking antibodies targeting B7‐H3/c‐Met interaction demonstrates promising therapeutic potential. This study expands the understanding of B7‐H3 biology and offers a new direction for targeted cancer treatments.

## 4 Materials and Methods

### 4.1 Patients and Tissue Samples

Tumor samples were obtained from 197 patients with CRC through surgical procedure and were confirmed by pathology department at the First Affiliated Hospital of Soochow University (Suzhou, China) between the period of 2004 and 2007. The clinic‐pathologic characteristics of the patients and the follow‐up details with different B7‐H3 distributions or c‐Met expression are described in Tables S1 and S3. Additionally, 10 more tissue samples were freshly isolated from the CRC patients at the same hospital over the year of 2014 to confirm B7‐H3 expression by western blotting and real‐time PCR analyses. Moreover, the expressions of B7‐H3 and c‐Met in the specimens were subsequently detected by immunohistochemical assay. All clinical samples were obtained with patients' informed consent and approved by the Ethics Committee of The First Affiliated Hospital of Soochow University (approval number: 2018121).

### 4.2 Cell Lines

The CRC cell lines HCT116 and SW480 were obtained from Cell Bank of Shanghai Institute for Biological Sciences, Chinese Academy of Sciences (Shanghai, China). The other four CRC cell lines (HT29, DLD1, RKO, and Colo205) were obtained from the International Joint Cancer Institute, The Second Military Medical University (Shanghai, China). All the cell lines were maintained in Dulbecco's modified Eagle's medium (DMEM; Invitrogen, Paisley, United Kingdom) supplemented with 1% penicillin plus streptomycin (Sigma‐Aldrich, St. Louis, MO), and 10% fetal bovine serum (FBS; Hyclone, Logan City, UT, USA). The GBM cell line U87MG (American Type Culture Collection, Rockville, MD) was cultured according to the supplier's instructions. All cell lines tested negative for Mycoplasma contamination.

### 4.3 Flow Cytometry

Cells were resuspended in PBS containing 1% FBS and stained with fluorescent‐conjugated antibodies against B7‐H3 (eBioscience, Hatfield, UK), CD133 (Miltenyi Biotec, Teterow, DE), CD44 (BD Biosciences, Oxford, UK), and CD166 (eBioscience) for 30 min at 4°C, protected from light. Specimens were subsequently analyzed by a Beckman Coulter FC500 Flow cytometer. Propidium iodide (10 µg/mL, Sigma‐Aldrich) was added immediately prior to acquisition to discriminate dead cells from viable cells. Data analysis was performed using FlowJo7.6 (Treestar Inc., San Carlos, CA).

### 4.4 Small Interfering RNA, Lentivirus, and Plasmids

The siRNA targeting specific human B7‐H3 sites (si996 and si1041), c‐Met site (siMet), and negative control siRNA (siNC) were synthesized by GenePharma (Shanghai, China). Details are shown in Table S4.

The lentiviral vectors encoding short hairpin RNAs targeting the human B7‐H3 or scramble shRNA were purchased from Sigma‐Aldrich and were designated as shB7‐H3 and shNon, respectively. Details are shown in Table S5. Furthermore, the lentiviral vectors encoding the human B7‐H3 gene were generated by using pLVX‐puro vector and were designated as B7‐H3. The empty vector was used as a negative control and was designated as EV. All the lentiviral vectors used in this study were synthesized by Genechem (Shanghai, China).

Moreover, plasmid EF.STAT3C.Ubc.GFP constitutively expressed active STAT3 was a gift from Linzhao Cheng (Addgene plasmid # 24983, Cambridge, MA, USA).

### 4.5 Immunoblotting (Western Blotting)

Protein was extracted from the cells using 1% SDS lysis buffer (62.5 mM Tris‐HCl, 2% w/v SDS, 10% glycerol, 50 mM DTT, 0.01% w/v bromophenol blue) supplemented with protease inhibitor cocktail (100×; Beyotime, Shanghai, People's Republic of China), resolved by SDS‐polyacrylamide gels and then transferred to PVDF membranes (0.45 µm, Millipore, Billerica, MA, USA). Primary antibodies against B7‐H3 (1:1,000, R&D Systems), c‐Met (1:1,000, Cell Signaling Technology), phospho‐Met (Tyr1234/1235, 1:1,000, Cell Signaling Technology), STAT3 (1:1,000, Cell Signaling Technology), phospho‐STAT3 (Tyr705, 1:1,000, Cell Signaling Technology), and GAPDH (1:10,000, MultiSciences, China) were used. HRP‐conjugated anti‐goat, anti‐rabbit, anti‐mouse IgG secondary antibodies were used. Finally, antigen–antibody reaction was visualized by the enhanced Pierce ECL Western blotting substrate kit (Thermo Scientific/Pierce, Rockford, IL, USA).

### 4.6 Real‐Time PCR

Isolation of total cellular RNA was carried out by using the NucleoSpin RNA kit (# 740955, MACHEREY‐NAGEL, Duren, Germany), and then the first‐strand cDNA was generated by using the PrimeScript RT reagent kit (# RR037A, Takara, Dalian, People's Republic of China). The real‐time PCR was performed using SYBR Premix Ex Taq II kit (# RR820L, TaKaRa, Dalian, People's Republic of China) according to the manufacturer's instruction. The primer sequences are listed in Table S6. Data were collected and analyzed with a CFX Connect Real‐Time PCR Detection System instrument (Bio‐Rad, CA, USA).

### 4.7 Co‐Immunoprecipitation Analysis

U87MG or SW480 cells were harvested in cell lysis buffer (# P0013, Beyotime) supplemented with protease inhibitor cocktail (# ST506, Beyotime, ×100). The cell lysate was immunoprecipitated with anti‐HA antibody (1:50, Cell Signaling Technology), separated by SDS‐PAGE and subjected to western blotting analysis with anti‐c‐Met antibody. Furthermore, the cell lysate was immunoprecipitated with anti‐c‐Met antibody (1:50, Cell Signaling Technology), separated by SDS‐PAGE and subjected to western blotting analysis with anti‐B7‐H3 HA.

### 4.8 Surface Plasmon Resonance

To determine the interaction between B7‐H3 and c‐Met, SPR was performed using a Biacore T200 instrument (GE Healthcare, Piscataway, NJ) according to the manufacturer's instructions. Briefly, the c‐Met‐his (Sino Biological, Beijing, China) protein was diluted with 10 mM sodium acetate (pH 4.5) to 200 µg/mL and immobilized on the CM5 sensor chip (GE Healthcare). Next, B7‐H3‐his (BioIntron, Taizhou, China) was analyzed at serial doubling concentrations maximized at 100 µM. Furthermore, the binding analyses were performed at a flow rate of 30 µL/min at 25°C. Finally, kinetic rate (KD) constants were calculated by fitting globally to the 1:1 model (one‐to‐one binding model) including in the Biacore T200 evaluation software v3.0.

### 4.9 Computer Structure Analysis

AlphaFold 3 is designed to predict the structure and interactions of the complex in the paper. All structural figures were prepared using the PyMOL.

### 4.10 Sphere Formation Assay

Tumor cells (1000 cells/mL) were cultured in ultralow attachment plates (Corning, NY, USA) with serum‐free DMEM (Invitrogen, Paisley, United Kingdom), containing 4 µg/mL insulin (Sigma‐Aldrich, St Louis, MO, USA), 20 ng/mL epidermal growth factor (EGF; PeproTech, Rocky Hill, NJ, USA), B27 (1:50, Invitrogen), and 0.4% bovine serum albumin (Sigma). The diameter of spheres >100 µm were counted after culturing for 14 days. Five replicate wells were included in each analysis and at least two independent experiments were conducted.

### 4.11 Cell Viability and Chemo‐Resistance Assays

Cell Counting Kit‐8 (CCK‐8, Dojindo, Japan) assay was used to determine the viability of the indicated cells. Briefly, 8000 cells were seeded into 96‐well plates and incubated overnight at 37°C. The cells were then treated with 5‐Fu or Cisplatin for different time points (24 h, 48 h). Thereafter, CCK‐8 solution was added to each individual well, and the plates were incubated for 4 h at 37°C. Finally, cell viability was determined by enzyme immunoassay analyzer (Bio‐Rad, CA, USA) according to the manufacturer's instructions. Five replicate wells were included in each analysis and at least two independent experiments were conducted.

### 4.12 Statistics

The measurements of all statistical values were performed using SPSS 22.0 software (Chicago, IL, USA) or Graphpad Prism 6.0. For parametric data, the *t* test was used to determine statistical significance. When comparing data sets between more than two groups, the one‐way ANOVA test was used. For correlation analyses, Pearson correlation test was used. For survival analyses, the Kaplan–Meier analysis was applied. Error bars in the experiments indicate standard error of the standard deviation (SD) or standard error of mean (SEM) for a minimum of two independent experiments. *p* values < 0.05 were considered statistically significant.

## Author Contributions

X.Z., B.L., G.Z., J.W., L.C., Y.X., and Y.H. conceived and designed the study. L.C., Y.X., Y.H, F.F., L.H., S.Z., and Y.F. performed experiments. X.H. completed the computational simulation analysis of protein–protein interaction structure. W.L. supervised construction of the manuscript. V.M. supervised the proteomics‐related experiments. X.Z., Y.I., H.L., H.M., K.‐O.J. supervised the experiments on GBM and the identification of protein interactions. X.Z., L.C., Y.X., and Y.H. interpreted the results. X.Z., W.L., L.C., Y.X., and Y.H. wrote the manuscript. All authors have read and approved the final manuscript.

## Conflicts of Interest

All authors declared no conflicts of interest.

## Ethics Statement

Informed consent was obtained from all participants in the study, and all related procedures were performed with the approval of the internal review and ethics boards of the First Affiliated Hospital of Soochow University. All animal experiments described in this study were conducted in accordance with the guidelines of the First Affiliated Hospital of Soochow University and were approved by the First Affiliated Hospital of Soochow University animal care and use committee (approval number: 2018121). Regarding cases where tumor volume in some mice exceeded 2000 mm^3^, we submitted a special ethical approval application from Ethics Committee of Soochow University (approval number: SUDA20250521A01) with the following justifications: The animal experiment can only be terminated when the expected experimental outcomes are achieved. Additionally, due to individual differences among mice within the experimental group, variations exist in their tumor‐forming response following tumor cell inoculation. Therefore, we typically conclude the experiment when 80% of the mice in the group exhibit tumor volumes ≤2000 mm^3^. Under these conditions, approximately 20% of the mice may show tumor volumes slightly exceeding 2000 mm^3^. To ensure scientific rigor in the experimental results, all mice in the experimental group will be included in the statistical analysis. This clarification is provided to emphasize methodological transparency.

## Supporting information



Supporting Information

## Data Availability

All CRC and GBM data for Figure S7A,B were obtained as Tier 1 “open‐access” files from the NIH Genomic Data Commons (official TCGA portal, https://www.cancer.gov/tcga), so neither dbGaP approval nor additional institutional clearance was required.
