# Correction to “Identification of c‐Met on Tumor Cells as a Novel Receptor for B7‐H3 Entails Implications for Cancer Cell Stemness and Targeted Therapy”

**DOI:** 10.1002/mco2.70416

**Published:** 2025-10-22

L. Cao, Y. Xu, Y. Hu, et al., “Identification of c‐Met on Tumor Cells as a Novel Receptor for B7‐H3 Entails Implications for Cancer Cell Stemness and Targeted Therapy,” *MedComm*, 6 (2025): e70332. https://doi.org/10.1002/mco2.70332.

Incorrect Affiliation: The second affiliation listed for the author “Yizhou Hu” is currently “Division of Molecular Neurobiology, Department of Medical Biochemistry and Biophysics, Karolinska Institute, Stockholm, Sweden”. This should be replaced to the correct second affiliation: “Department of Laboratory Medicine, Karolinska Institute, Stockholm, Sweden”. The first affiliated unit is correct and requires no modifications.

During the final submission of the manuscript (Manuscript ID: MCO270332), an error occurred in the author list. Professor Marko Hyytiäinen was erroneously omitted. His name is now correctly reinstated as a co‐author to reflect his substantial contributions. Professor Marko Hyytiäinen made substantial intellectual contributions to this work, which fully satisfy the authorship criteria as defined by the International Committee of Medical Journal Editors (ICMJE): Study Conception and Design: Professor Hyytiäinen provided critical input during the initial conception of the research project and contributed significantly to the development of the study design. Acquisition of Critical Data: He was directly involved in and supervised the acquisition of key experimental data, ensuring its robustness and validity. Drafting and Critical Revision of the Manuscript: Professor Hyytiäinen participated in revising the manuscript critically for important intellectual content across several drafts, substantially improving its scholarly quality and clarity.

Marko Hyytiäinen, Afffiliation: Research Program, Faculty of Medicine, University of Helsinki, and The Hospital District of Helsinki and Uusimaa, Helsinki, Finland

The Acknowledgements section remains unchanged.

We apologize for this error.

