# Supplementary material for: Identification of c‐Met on Tumor Cells as a Novel Receptor for B7‐H3 Entails Implications for Cancer Cell Stemness and Targeted Therapy
Source: MedComm (2020). 2025 Aug 22;6(9):e70332. doi: 10.1002/mco2.70332 (PMC12371199; doi:10.1002/mco2.70332)
Supplement: Supplementary file 1 — Supporting Information [file MCO2-6-e70332-s001.docx]

**Identification of c-Met on tumor cells as a novel receptor for B7-H3 entails implications for cancer cell stemness and targeted therapy**

Lei Cao^1#^, Yunyun Xu^2#^, Yizhou Hu^3,4#^, Xue Huang^1#^, Fengqing Fu^1,5^, Shenghua Zhan^1^, Lili Huang^2^, Yangyang Feng^6^, Ylivinkka Irene^3^, Huini Li^3^, Varjosalo Markku^7^, Keski-Oja Jorma^3^, Guangbo Zhang^1^, Binfeng Lu^8^, Jian Wang^2^, Wanli Liu^6^*, Xueguang Zhang^1,5,9^*

1. Jiangsu Institute of Clinical Immunology & Jiangsu Key Laboratory of Clinical Immunology, The First Affiliated Hospital of Soochow University, Suzhou, China.
2. Institute of Pediatrics, Children’s Hospital of Soochow University, Suzhou, China.
3. Research Program, Faculty of Medicine, University of Helsinki, and The Hospital District of Helsinki and Uusimaa, Helsinki, Finland.
4. Division of Molecular Neurobiology, Department of Medical Biochemistry and Biophysics, Karolinska Institute, Stockholm, Sweden.
5. State Key Laboratory of Radiation Medicine and Protection, Soochow University, Suzhou, China.
6. MOE Key Laboratory of Protein Sciences, Center for Life Sciences, School of Life Sciences, Beijing Key Lab for Immunological Research on Chronic Diseases, Institute for Immunology, Tsinghua University, Beijing, China.
7. Institute of Biotechnology and Helsinki Institute of Life Science, University of Helsinki, Helsinki, Finland.
8. Department of Immunology, School of Medicine, University of Pittsburgh, Pittsburgh, USA.
9. Soochow University-Bright Scistar Antibody Joint Laboratory, Suzhou, China.

# These authors contributed equally.

*Corresponding author. E-mail: [xgzh@suda.edu.cn](mailto:xgzh@suda.edu.cn); [liulab@tsinghua.edu.cn](mailto:liulab@tsinghua.edu.cn)

**Supplementary information for Identification of c-Met on tumor cells as a novel receptor for B7-H3 entails implications for cancer cell stemness and targeted therapy**

**Supplementary Materials and Methods**

**Immunohistochemistry**

Hematoxylin and eosin (HE) staining and immunohistochemistry were performed on paraffin-embedded tissues using standard protocols. In brief, paraffin-embedded tumor tissues and their corresponding nearby non-tumor colorectum were sectioned at 4 μm thickness. To remove aldehyde links formed during initial fixation of tissues, antigen retrieval had to be performed by a pressure cooker for 3 min in 0.01 M citrate buffer (pH 6.0). Moreover, histological sections were separately incubated with antibodies specific for B7-H3 (1:100, R&D Systems, Abingdon, UK), c-Met (1:400, Cell Signaling Technology, Danvers, MA, USA) overnight at 4 ºC. The immunodetection was performed on the following day using DAB (Dako, Carpinteria, CA) according to the manufacturer’s instructions. Finally, immunostaining scores were independently conducted by three experienced pathologists according to staining intensity. Tumor tissues from HCT116 xenografts of mice treated with 10 mg/kg 3E8 mAb or IgG for 4 weeks were dissected and fixed in 10% buffered formalin. According to the standard protocols, paraffin-embedded sections were then stained with anti-human B7-H3 (1:100, R&D Systems), anti-human CD133 (1:200, Abcam, Cambridge, MA, USA). Stained slides were imaged using the microscopy (Nikon Eclipsc Ni, Tokyo, Japan).

**Immunofluorescence**

Paraffin-embedded samples were sequentially treated with antigen retrieval and blocked in PBS containing 3% bovine serum albumin (BSA) according to the methods used in the ‘**Immunohistochemistry**’. After, samples were incubated with primary antibodies specific for B7-H3 (1:100, R&D Systems), c-Met (1:400, Cell Signaling Technology), Bmi1 (1:200, Cell Signaling Technology), CD133 (1:200, Abcam) and Sox2 (1:100, Santa Cruz) overnight at 4 ºC. Moreover, incubation of Horseradish peroxidase (HRP)-conjugated secondary antibodies (Life technologies, Duren, DE) and TSA^TM^ System dyes (# 2336651, Fluorescein System; # 2384212, Cyanine 3 System, PerkinElmer, MA, USA) were separately carried out for 30 min and 10 min at room temperature. Finally, DAPI (Dojindo Laboratories, Kumamoto, Japan) was then used for counterstaining the nuclei and images were obtained by a fluorescent microscopy (Nikon Eclipsc Ni, Tokyo, Japan) or laser scanning confocal microscopy (OLYMPUS IX83, Tokyo, Japan). Additionally, indirect immunofluorescence detecting cell lines was performed. Cells were cultured in a 24-well plate and when they were confluent to 60–70%, washed with PBS for three times, fixed with 4% paraformaldehyde and permeabilized in 0.1% Triton X-100 (Sigma, MO) for 30 min at 4 ◦C. After washing three times and blocking with 5% BSA, cells were incubated with B7-H3 and c-Met overnight at 4 ◦C. Cells were washed three times and incubated with goat anti-mouse Alexa Fluor 594 and goat anti-rabbit Alexa Fluor 488 (Thermo Fisher Scientific, CA) for 30 min in the dark. Finally, localization of B7-H3 and c-Met was photographed using confocal microscopy with identical exposure times.

**Tandem affinity purification and mass spectrometry**

Strep-HA pcDNA3 was kindly provided by Dr. Markku Varjosalo, and double purifications were described in previous study with some modifications [1]: U87MG cells expressing Strep/HA tagged B7-H3 were grown to 70% confluency and were then lysed with RIPA buffer (50 mM Tris pH 7.4, 150 mM NaCl, 0.1% SDS, 1% NP-40, 0.1% SDS) containing complete protease inhibitors (Roche). Cell lysates were incubated with StrepTactin Sepharose (GE) on an end-over-end shaker at 4 °C for 2 to 4 h. After incubation, sepharose beads were collected by centrifugation and washed 3 times with the lysis buffer. After washing, the proteins were eluted from the sepharose beads by incubation with 1 mL 10 mM biotin for 5 min. The eluates were then incubated with anti-HA sepharose beads in an end-over-end rotating shaker for 1 h. After incubation, the suspension was transferred to a Bio-Spin mini-chromatography column (Bio-Rad, Hercules, USA) and the sepharose beads were washed 3 times with the lysis buffer. The proteins were eluted by incubating the sepharose beads with 500 μL 0.1 M glycine PH 2.4. Subsequently the elution was neutralized with 100 μL 1 M Tris PH 8.0. The eluted proteins were validated by immunoblotting and visualized by silver staining, and the resulting peptides were identified by mass spectrometry analysis.

**Proximity Ligation Assay (PLA)**

PLA was used to identify specific protein-protein interactions through oligonucleotide-conjugated secondary antibodies. The assay was performed according to the manufacturer's protocol with the Duolink® In Situ Red Starter Kit Mouse/Rabbit (In situ Proximity Ligation Assay reagent; Sigma-Aldrich, USA). In brief, cells were fixed with 4% paraformaldehyde and then permeabilized using 0.2% Triton X-100. After that, the cells were incubated with a blocking solution for 60 minutes, followed by incubation with primary antibodies (B7-H3, 1:200, Proteintech; c-Met, 1:1500, Cell Signaling Technology) at 4°C overnight. Next, the cells were treated with secondary antibodies conjugated to oligonucleotides (anti-rabbit PLUS probe and anti-mouse MINUS probe) at 37°C for 60 minutes, followed by incubation with the ligation-ligase solution at 37°C for 60 minutes, and amplification with the polymerase solution at 37°C for an additional 100 minutes. Finally, counterstaining of the nuclei was performed using DAPI, and images were acquired using the Mantra Snap and inform software (Akoya Biosciences). Every distinct red fluorescent spot represents the close proximity of two interacting proteins within the cells.

**Generation and Characterization identify of anti-human B7-H3 mAbs**

Hypoxanthineaminoperin-thymidine (HAT) and hypoxanthine-thymidine (HT) products were from Sigma (St. Louis, MO). CHO (Chinese hamster ovary) cells and mouse myeloma cells (SP2/0 cell line) were obtained from Ameican Type Culture Collection (ATCC, Manassas, VA). CHO/Mock cells (CHO cells transfected with empty vector) and CHO/B7-H3 cells (CHO cells transfected with human B7-H3 genes) were from our lab (Jiangsu Institute of Clinical Immunology, Suzhou, China). RPMI-1640 media supplemented with 10% fetal bovine sera (FBS) were used for cell culture. BALB/c mice were purchased from Shanghai Lab Animal Research Center (Shanghai, China). The institutional protocol for animal studies was approved by the Institutional Animal Care and Use Committee of the First Affiliated Hospital of Soochow University (approval number: 2018121).

CHO/B7-H3 cells were used as immunogens. Female BALB/c mice (6–8 weeks old) were immunized with CHO/B7-H3 cells (1 × 10^7^/mouse) pretreated by mitomycin (0.5 μg/μl). The booster injection was repeated three times every 21 days. Three days after the final booster immunization, the splenocytes from selected mice were harvested and fused with the SP2/0 cell line (ATCC, Manassas, VA) in the presence of 50% polyethylene glycol (PEG). The fusion cells were cultured with DMEM medium containing HAT and 15% FBS in 96-well plates to select hybrid clones. The hybrid clone supernatants were screened for the detection of antibody by enzyme-linked immunosorbent assay (ELISA) and flow cytometry. The hybrid clone-secreting antibodies recognized CHO/B7-H3 cells but not CHO/Mock cells. The positive clones were subcloned to establish hybridoma cell lines that secreted momoclonal antibody. Protein G Sepharose affinity column (GE, Sweden) was used to purify the ascites antibodies.

Ig isotypes were identified by Mouse Monoclonal Antibody Isotyping Kit (Roche, Mannheim, Germany) according to the manufacturer’s instructions. The specificity and reactivity of antibodies was investigated by flow cytometric analysis. CHO/B7-H3 cells were incubated with mAbs for 30 min at 4 ◦C. After washing for three times, the cells were resuspended in goat anti-mouse antibody conjugated with PE. Cells were incubated at 4 ◦C for another 30 min, followed by washed twice and finally analyzed using flow cytometry. Meanwhile, CHO/B7-H3 cells were directly stained with the commercial PE-conjugated mouse anti-human B7-H3 antibody as a positive control or human IgG as a negative control.

Determination of the binding ability of the obtained mAbs to immobilized B7-H3Ig antigen was by indirect ELISA. Briefly, the recombinant B7-H3Ig fusion protein diluted to different concentrations (100, 50, 25, 12.5, 6.25, 3.125, 1.5625 and 0 ng/ml) was added to each well of an ELISA plate and incubated at 4 ◦C overnight. After extensive washing with PBS containing 0.1% Tween-20 (PBST), each well was blocked with 3% bovine serum albumin (BSA) for 1 h at 37 ◦C. Then the obtained antibodies (1 mg/ml) were added for 2 h at room temperature, followed by HRP-goat anti-mouse IgG at 1:8000 for 1 h. After washing with PBST for 10 times, the plate was incubated with tetramethylbenzidine (TMB, Sigma, MO) and stopped by 2 M sulfuric acid. The absorbance at 450 nm was determined with a microplate reader (Bio-Rad, Hercules, CA).

A competition assay was performed to determine whether the antibodies could recognize different epitopes of B7-H3 antigen. CHO/B7-H3 cells were incubated with an unlabeled anti-B7-H3 mAb for 30 min at 4 ◦C, then the cells were washed and stained with another biotinylated anti-B7-H3 mAbs followed by streptavidin-PE for 30 min at 4 ◦C. The results were analyzed by flow cytometry. CHO/B7-H3 cells were also stained directly with biotinylated mouse IgG followed by streptavidin-PE as a negative control or biotinylated anti-B7-H3 mAbs as positive control.

Western blotting was used to show the antibodies specifically recognized the transfected cells, CHO/B7-H3, but not CHO/Mock. Recombinant B7-H3Ig or CHO/B7-H3 cells containing high levels of B7-H3 were separated on 10% non-reducing gels. CHO/Mock and IgG protein were used as negative controls. After transferring the membrane, washing with PBST and blocking with 5% BSA, the target bands were stained with mAbs followed by HRP-labeled goat anti-mouse IgG mAb (Affinity Biosciences, OH). The membranes were visualized with Super ECL Reagents (Applygen Technologies Inc., Beijing). Commercial B7-H3 antibody was used as a positive control.

**Enzyme Linked Immunosorbent Assay (ELISA)**

Cells were cultured in DMEM with 10% FBS until 80% of confluency. Then, cells were washed with PBS and cultured in fresh serum-free media. After 24 h, supernatants were harvested for subsequent ELISA. The B7-H3 ELISA kits were purchased from R&D Systems. All experiments were performed according to the manufacturer’s instructions.

To examine B7-H3 and c-Met interaction, 96-well ELISA plates were coated with 5 μg/mL of recombinant c-Met-his (Sino Biological). Then serial dilutions of B7-H3-biotin or PD-L1-Biotin fusion proteins (BioIntron) were added and incubated for 1 h at 37 °C. Furthermore, B7-H3-biotin was also added to PD-1-his–coated plates as negative control. The streptavidin-HRP (Multi Sciences) at 1:10,000 was used for detection. The substrate for HRP was TMB using the microwell peroxidase substrate system (KPL).

To determine if c-Met can bind to B7-H3 and HGF simultaneously, 96-well ELISA plates were coated with 5 μg/mL of recombinant c-Met-his as above. Then B7-H3-biotin fusion proteins at 5 μg/mL were preincubated with HGF (# 100-39, PeproTech, NJ, USA), TNF-α (# 300-01A, PeproTech) or IFN-γ (# 300-02, PeproTech) at serial dilutions for 15 min at room temperature, and then added to the plates and incubated for 1 h at 37 °C. Here TNF-α and IFN-γ were used as negative control.

To test the capacities of c-Met antibodies (α-c-Met) to block c-Met binding to B7-H3, 96-well ELISA plates were coated with 5 μg/mL of recombinant c-Met-his as above. Then B7-H3-biotin fusion proteins at 5 μg/mL were preincubated with α-c-Met (# AF276, R&D Systems) or isotype control IgG at serial dilutions for 15 min at room temperature, and then added to the plates and incubated for 1 h at 37°C. Here IgG were used as negative control.

To test the capacities of B7-H3 antibodies to block B7-H3 binding to c-Met, 96-well ELISA plates were coated with 5 μg/mL of recombinant c-Met-his as above. The 35 anti-B7-H3 mAbs independent-developed or isotype controls at the indicated concentrations were preincubated with 5 μg/mL B7-H3-biotin (R&D Systems) for 15 min, then added to the plates and incubated for 1 h at 37 °C. Streptavidin-HRP at 1:10,000 was used for detection. Note the anti-B7-H3 mAbs 3E8 and 4H3 were validated. Therefore, serial dilutions of 3E8, 4H3 mAbs or isotype controls were added and incubated with B7-H3-biotin as above. Finally, the blocking capacities of 3E8 and 4H3 mAbs were detected by streptavidin-HRP.

To compare the binding site between mAbs 3E8 and 4H3, a competition ELISA was performed. 10 µg/mL IgG, or 3E8 or 4H3 mAb were added to B7-H3-his coated (10 µg/mL) protein, after washing, add 1 µg/mL 3E8-Biotin to the IgG and 4H3 mAb wells and add 1 µg/mL 4H3-Biotin to the IgG and 3E8 mAb wells. The binding was detected by streptavidin-HRP.

**Animal Experiments**

4~6 week Male SCID mice and athymic BALB/c nude mice were purchased from the Shanghai Experimental Animal Center of Chinese Academic of Sciences (Shanghai, People’s Republic of China) and were maintained under defined conditions at the Animal Experiment Center of Soochow University. All animal experiments were approved by the Animal Care and Use Committee of Soochow University.

**Animal** **xenograft tumor:** For the establishment of xenograft tumors, 1×10^6^ of B7-H3 knockdown or control HCT116 cells, 3×10^6^ of U87MG cells, 2×10^6^ of NCI-N87 cells were suspended at a 1:1 ratio in 200 μL of complete medium and Matrigel (BD Biosciences), and delivered via subcutaneous injections into BALB/c nude mice as previously described [2]. The size and incidence of subcutaneous tumors were measured and recorded on a weekly basis. Then, tumor formation was observed for the following 11 weeks.

**Inhibitor treatment:** 5×10^6^ HCT116 cells with knocked down of B7-H3 were exogenously expressed with either empty vector (EV) or B7-H3 were implanted into 6-weeks-old BALB/c nude mice. One week after the subcutaneous injections, the inhibitor of c-Met (Crizotinib (PF-02341066), Selleck Chemicals, Houston, TX, USA) was given an intragastric administration at a dose of 25 mg/kg every other day. Then, tumor size was measured every week with a caliper, and the volume was calculated using the standard modified formula volume (mm^3^) = (length×height^2^) × π/6. Moreover, Cryptotanshinone (inhibitor of STAT3, Selleck Chemicals, Houston, TX, USA) administration was started at a dose of 50 mg/kg intraperitoneal injection (i.p.) every other day in mice subcutaneously injected with B7-H3 overexpressed or control HCT116 cells after 1 week. Then, tumor formation was assessed for up to 40 days.

**B7-H3 mAbs 3E8, 4H3 and 4G4 therapy:** 5×10^6^ HCT116 cells with knocked down of B7-H3 were exogenously expressed with either EV or B7-H3. These cells were then implanted into 6-weeks-old BALB/c nude mice. The administration of B7-H3 blocking antibodies (3E8, 4H3 or 4G4) were started at a dose of 10 mg/kg or 20 mg/kg i.p. every three days. Then, tumor size was measured every three days with a caliper, and the volume was calculated using the standard modified formula volume (mm^3^) = (length×height^2^) × π/6. Finally, tumor formation was assessed for up to 37 days.

**Combined therapy:** The combined treatment of B7-H3 blocking antibody 3E8 and c-Met inhibitor (Crizotinib, PF-02341066) was performed when the longest diameter of xenografts reached 5 mm. Crizotinib was administrated via intragastric administration every other day and 3E8 mAb (10 mg/kg) were injected by intraperitoneal injection every three days. Then, tumor size was measured every three days with a caliper, and the volume was calculated using the standard modified formula volume (mm^3^) = (length×height^2^) × π/6.

**Reference**

1. Varjosalo, M., Keskitalo, S., Van Drogen, A., Nurkkala, H., Vichalkovski, A., Aebersold, R., and Gstaiger, M. 2013. The protein interaction landscape of the human CMGC kinase group. *Cell Rep* 3:1306-1320.

2. Jia, R.J., Cao, L., Zhang, L., Jing, W., Chen, R., Zhu, M.H., Guo, S.W., Wu, G.B., Fan, X.Y., Wang, H., et al. 2014. Enhanced myeloid differentiation factor 88 promotes tumor metastasis via induction of epithelial-mesenchymal transition in human hepatocellular carcinoma. *Cell Death Dis* 5:e1103.

**Supplementary Figures**

**Figure S1 B7-H3 highly expressed in CRC tissues and cell lines**


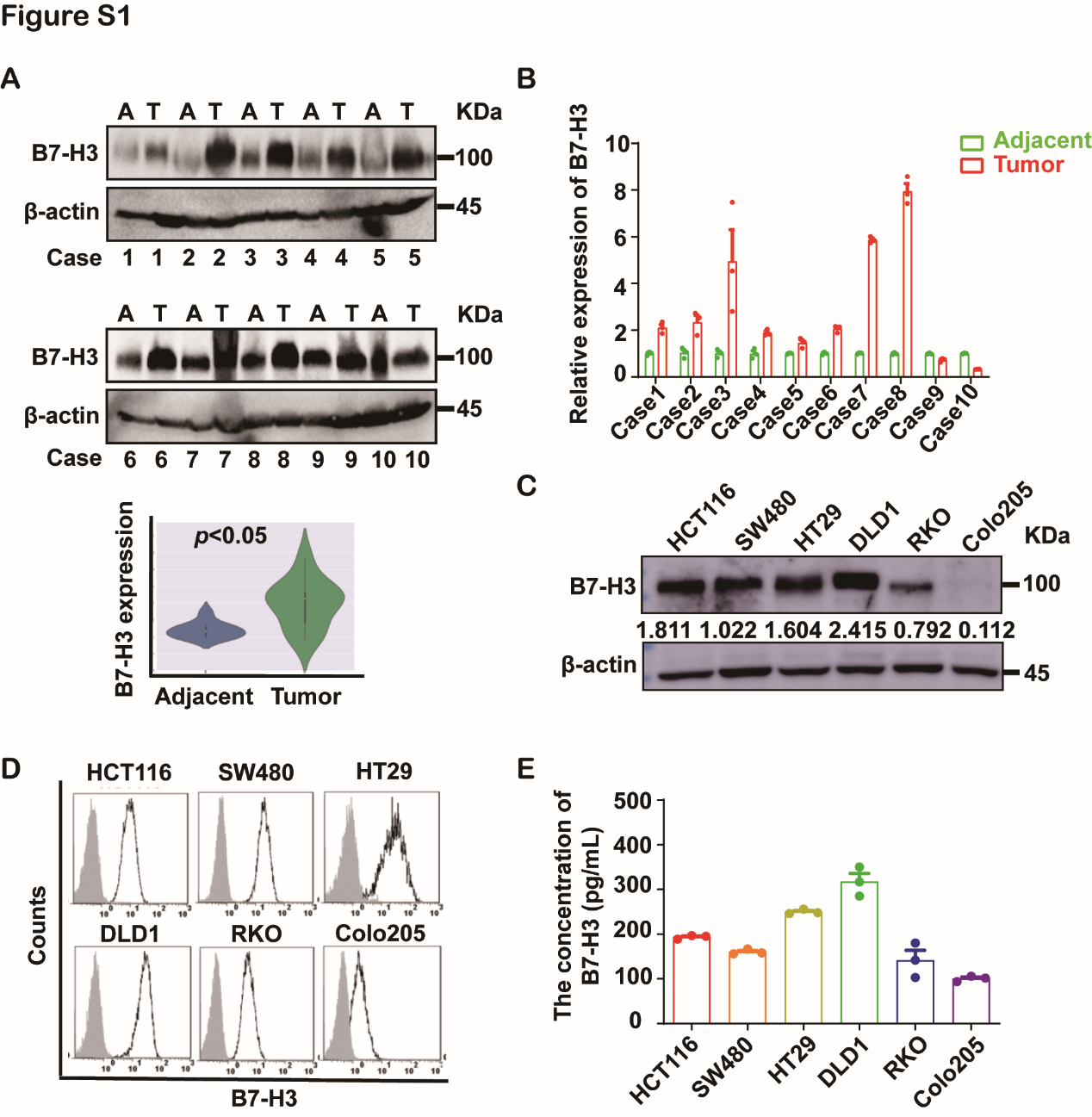


(A) Immunoblotting for B7-H3 in resected CRC tumor tissue (T), and paired tumor-adjacent normal tissue (A) (n = 10). β-actin was used as a loading control. Eight of ten cases exhibit higher expression of B7-H3 protein in tumor tissue. Violin plot at the right side indicates the relative expression of B7-H3 (tumor tissue, green; tumor-adjacent normal tissue, blue).

(B) Representative Real-time PCR for B7-H3 in resected CRC tumor tissue (T), and paired tumor-adjacent normal tissue (A) (n = 10). Eight of ten cases exhibit higher expression of B7-H3 mRNA in tumor tissue. Error bars denote SD.

(C) Immunoblotting analysis of B7-H3 expression in six CRC cell lines. All cell lines express B7-H3 protein. The β-actin was used as a loading control.

(D) Flow cytometric analysis of membrane-bound B7-H3 expression in six CRC cell lines (HCT116, SW480, HT29, DLD1, RKO, Colo205). All cell lines express membrane-bound B7-H3 at a decent level.

(E) ELISA analysis of soluble B7-H3 expression in the CRC cell lines. Error bars denote SD.

**Figure S2 B7-H3 regulates p-Stat3 via p-Met**


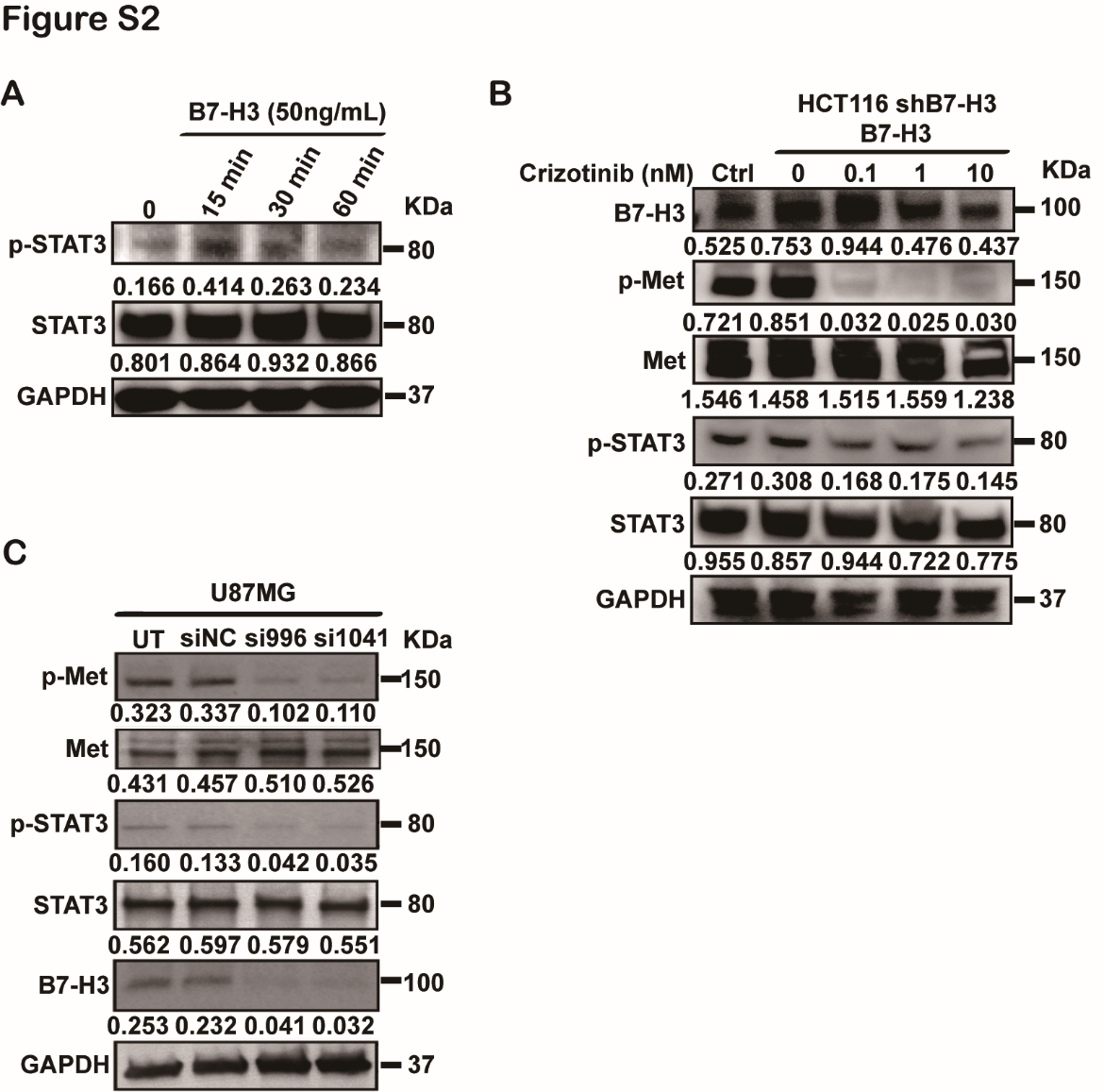


(A) Recombinant B7-H3 was incubated with HCT116 cells at the concentration of 50 ng/mL for 15 min, 30 min, or 60 min respectively. The levels of p-STAT3 and STAT3 in HCT116 cells were then determined by immunoblotting.

(B) The phosphorylation of c-Met in B7-H3 silenced HCT116 cells was rescued by exogenous expression of B7-H3, but inhibited by c-Met inhibitor crizotinib. The levels of B7-H3, c-Met, STAT3, p-Met and p-STAT3 in HCT116 cells were determined by immunoblotting.

(C) B7-H3 silencing impaired the phosphorylation of c-Met and STAT3 in U87MG cells. U87MG cells expressing B7-H3 siRNAs or control siRNA were cultured to 50% confluence, followed by immunoblotting. Phosphorylation of c-Met and STAT3 were inhibited in U87MG cells expressing B7-H3 specific siRNAs (si996 and si1041). GAPDH was used as loading controls.

**Figure S3 B7-H3 regulates tumor stemness upon c-Met and Stat3 signal**


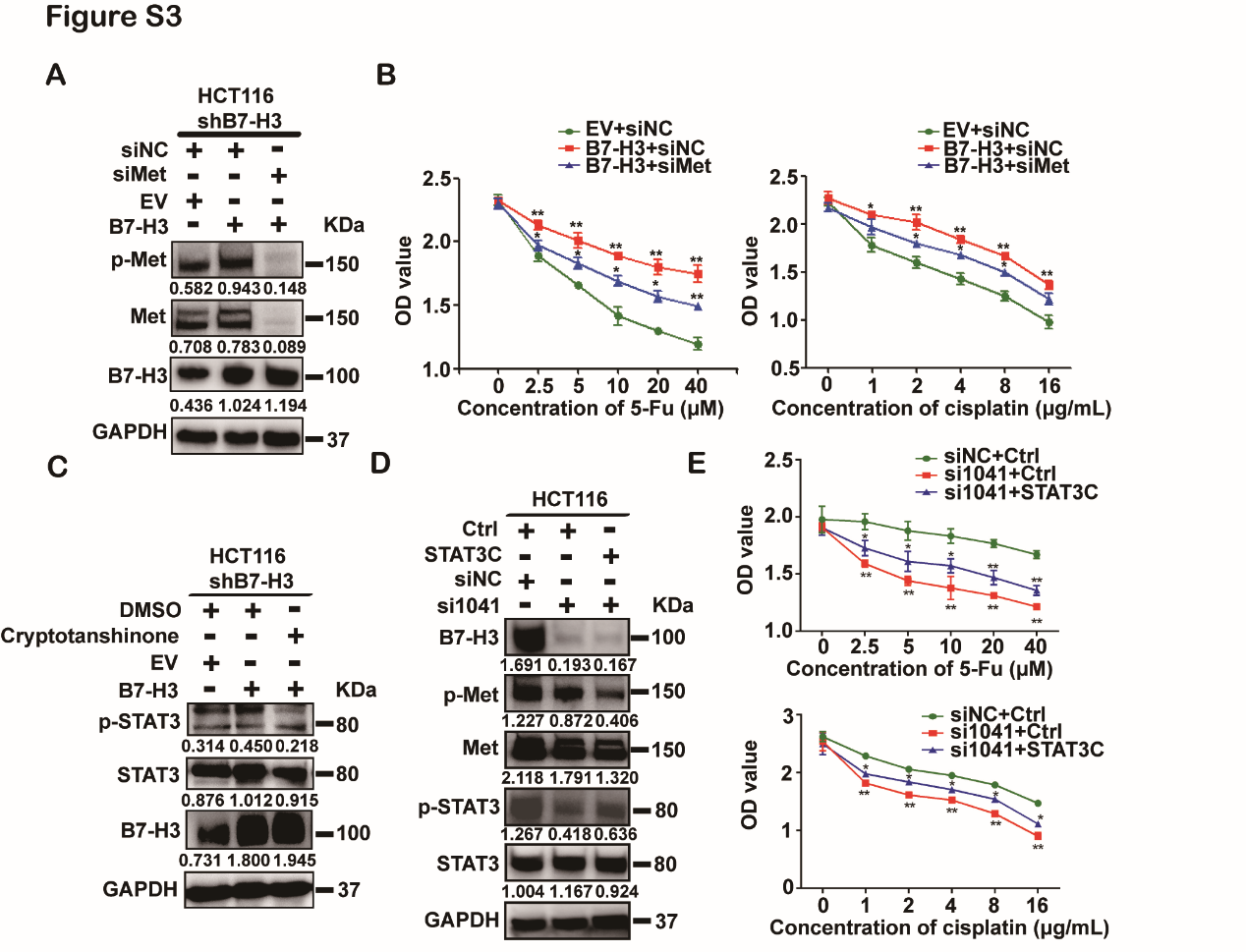


(A, B) Silencing c-Met attenuated the phenotype of the enhanced tumor stemness upon B7-H3 overexpression. B7-H3 was silenced by shRNA in HCT116 cells. B7-H3 levels were restored by lentivirus-based overexpression (empty vector, EV, was used as a control). As indicated in the combinational chart, these cells were then induced for c-Met knockdown by c-Met specific siRNA with siNC as a control. The protein levels of B7-H3, c-Met, and the phosphorylation levels of c-Met in the indicated types of HCT116 cells were determined by immunoblotting. GAPDH was used as a loading control. Bar chart represents the results of quantification. As indicated in the combinational chart (A), the cells as described in (B) were treated by chemotherapeutic drugs, 5-Fu (left) or cisplatin (right), followed by viability assay. X-axis represents the concentration; Y-axis represents the OD value.

(C) In B7-H3 silenced HCT116 cells, the phosphorylation of STAT3 was rescued by the overexpression of B7-H3, while this effect was attenuated by cryptotanshinone treatment. B7-H3 was silenced by shRNA in HCT116 cells. B7-H3 levels were restored by lentivirus-based overexpression (empty vector, EV, was used as a control). B7-H3, STAT3 and p-STAT3 levels were assessed by immunoblotting. GAPDH was used as a control.

(D) In B7-H3 silenced HCT116 cells, the transfection of STAT3C promoted STAT3 activation. The levels of B7-H3, c-Met, STAT3, p-Met and p-STAT3 were determined by immunoblotting. GAPDH was used as a control.

(E) B7-H3 was silenced by siRNA1041 in HCT116 cells, and siNC was used as a non-targeting control. The transfection of STAT3C was used to promote STAT3 activation. After the treatment of the chemotherapeutic drugs 5-Fu and cisplatin, the cells were applied for viability assay, and the results were illustrated in line chart. X-axis represents the concentration; Y-axis represents the OD value.

In (B) and (E), one-way ANOVA was used for statistical analysis, **, *P* value < 0.01, *, *P* value < 0.05, n >= 3/group, Error bars denote SD.

**Figure S4 B7-H3 mAbs were identified to block B7-H3-c-Met interaction**


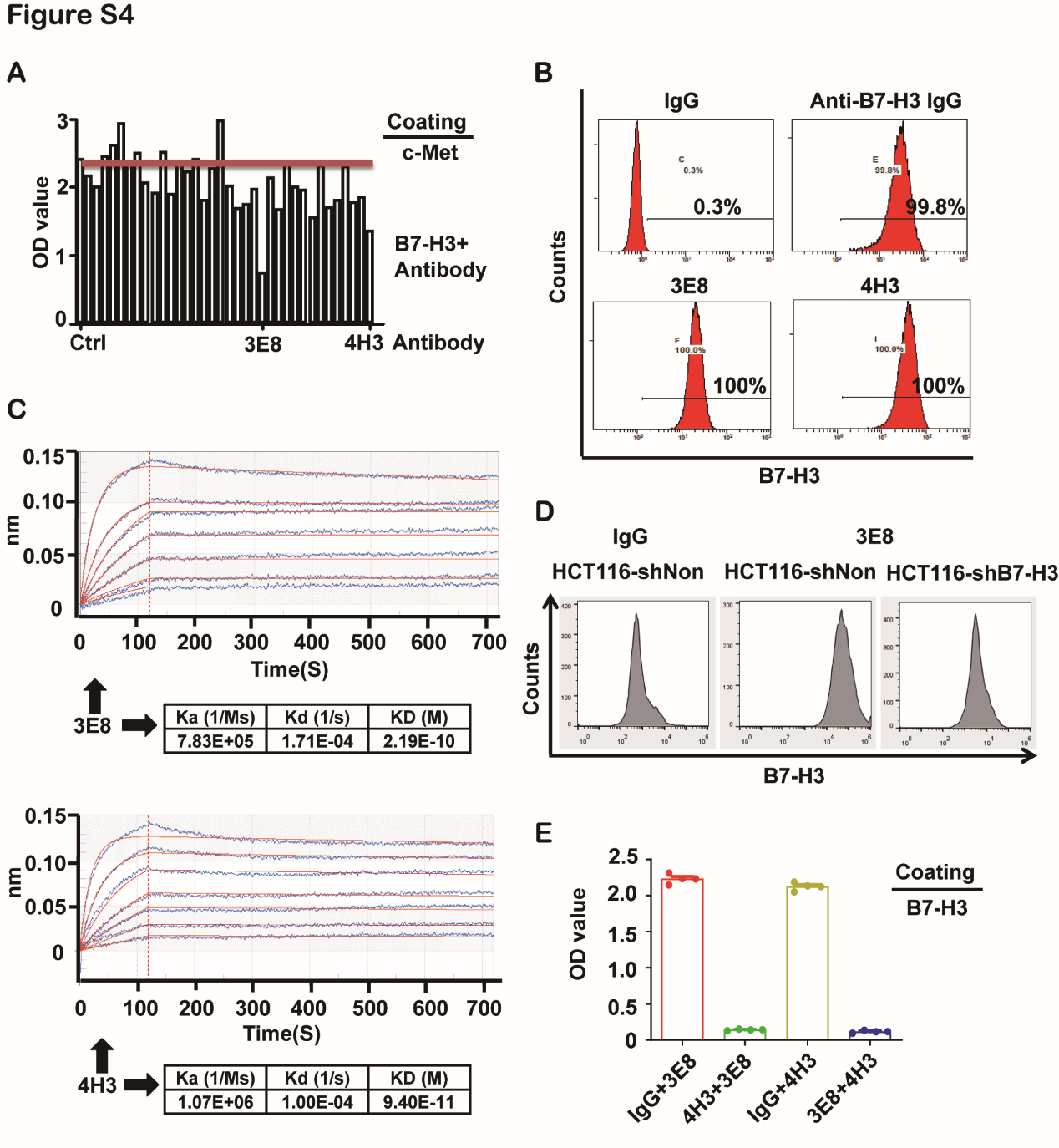


(A) The blocking capacities of B7-H3 mAbs against B7-H3 and c-Met interaction were determined by ELISA. B7-H3 recombinant protein (5 µg/mL) was pre-incubated with a panel of anti-B7-H3 mAbs (20 µg/mL). Then the mixture was added to c-Met pre-coated (5 µg/mL) plates. The binding was determined by streptavidin-HRP.

(B) FACS validated the recognition of B7-H3 by 3E8 or 4H3 mAb. B7-H3 overexpressed cells were stained by commercial available anti-B7-H3 IgG polyclonal antibody, 3E8 or 4H3 mAb respectively.

(C) Biolayer interferometry assessed the binding affinity of 3E8 (top panel) or 4H3 (bottom panel) mAb to B7-H3.

(D) FACS validated the recognition of B7-H3 in HCT116 shNon and HCT116 shB7-H3 cells by 3E8.

(E) Competition ELISA for the binding of B7-H3 to either 3E8 or 4H3 mAb as indicated in the combinational chart. The binding was detected by streptavidin-HRP. n >= 3/group. Error bars denote SD.

**Figure S5 The B7-H3-c-Met blocking antibody 3E8, 4H3 inhibits tumor growth**


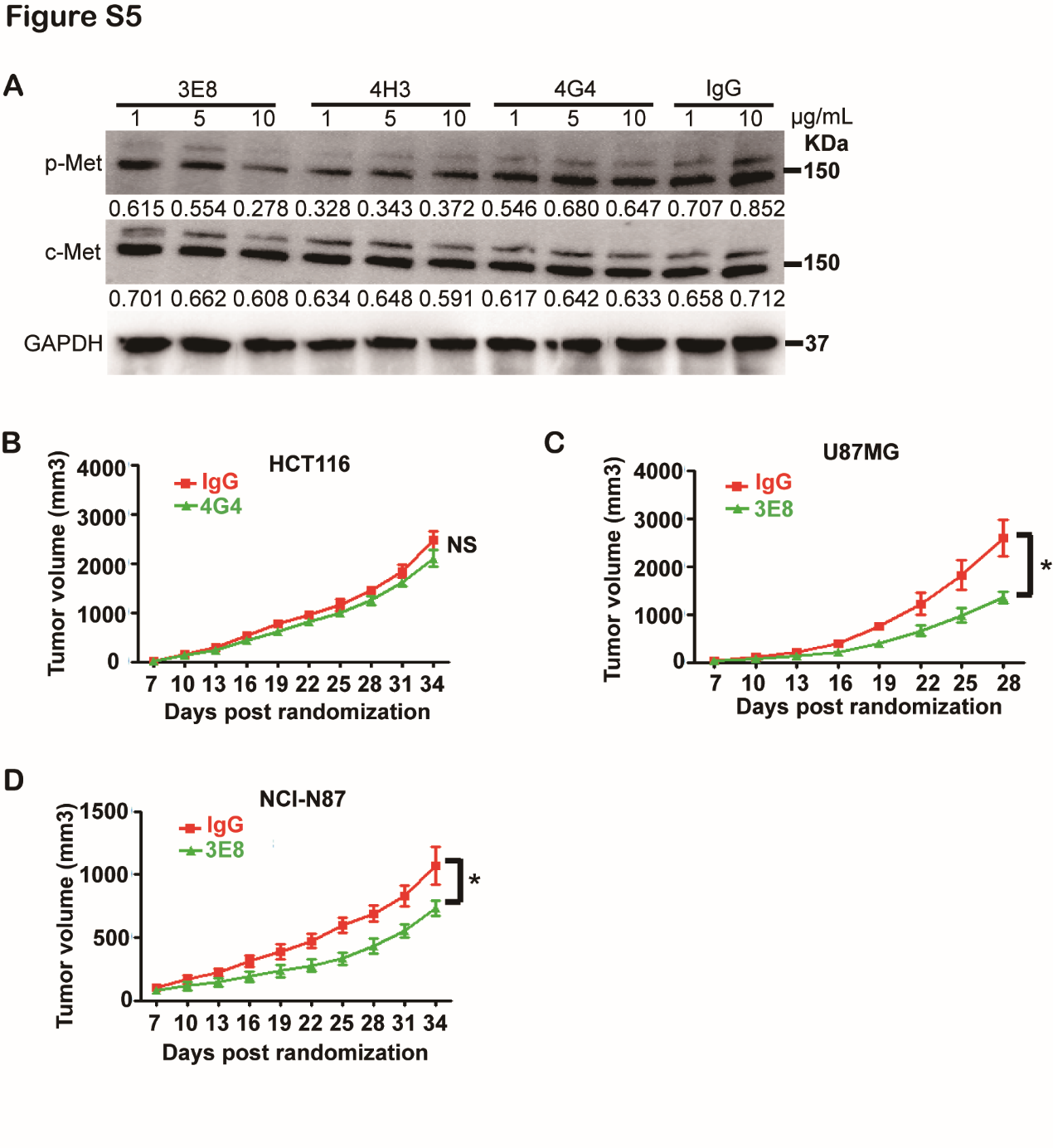


(A) HCT116 cells were pre-treated with the 3E8, 4H3 and 4G4 mAbs at the indicated concentrations in vitro, and p-Met, c-Met levels were evaluated by immunoblotting. IgG antibody was used as a negative control, and GAPDH as a loading control. Band intensities were quantified and indicated.

(B) Subcutaneous growth of colon HCT116 tumor cells in mice treated with 4G4 mAb (10 mg/kg) or IgG control. The tumor volumes throughout 34 days were marked in both groups.

(C) and (D) Subcutaneous growth of GBM U87MG (D) and gastric cancer NCI-N87 (E) tumor cells in mice treated with 3E8 mAb (10 mg/kg) or IgG control. The tumor volumes throughout 28 or 34 days were marked in both groups.

In (B)-(D), statistical analysis was performed using one-way ANOVA. *, P value < 0.05, n = 5 mice per group. Error bars denote mean ± SEM.

**Figure S6 Residue annotation diagrams for the predicted binding sites**


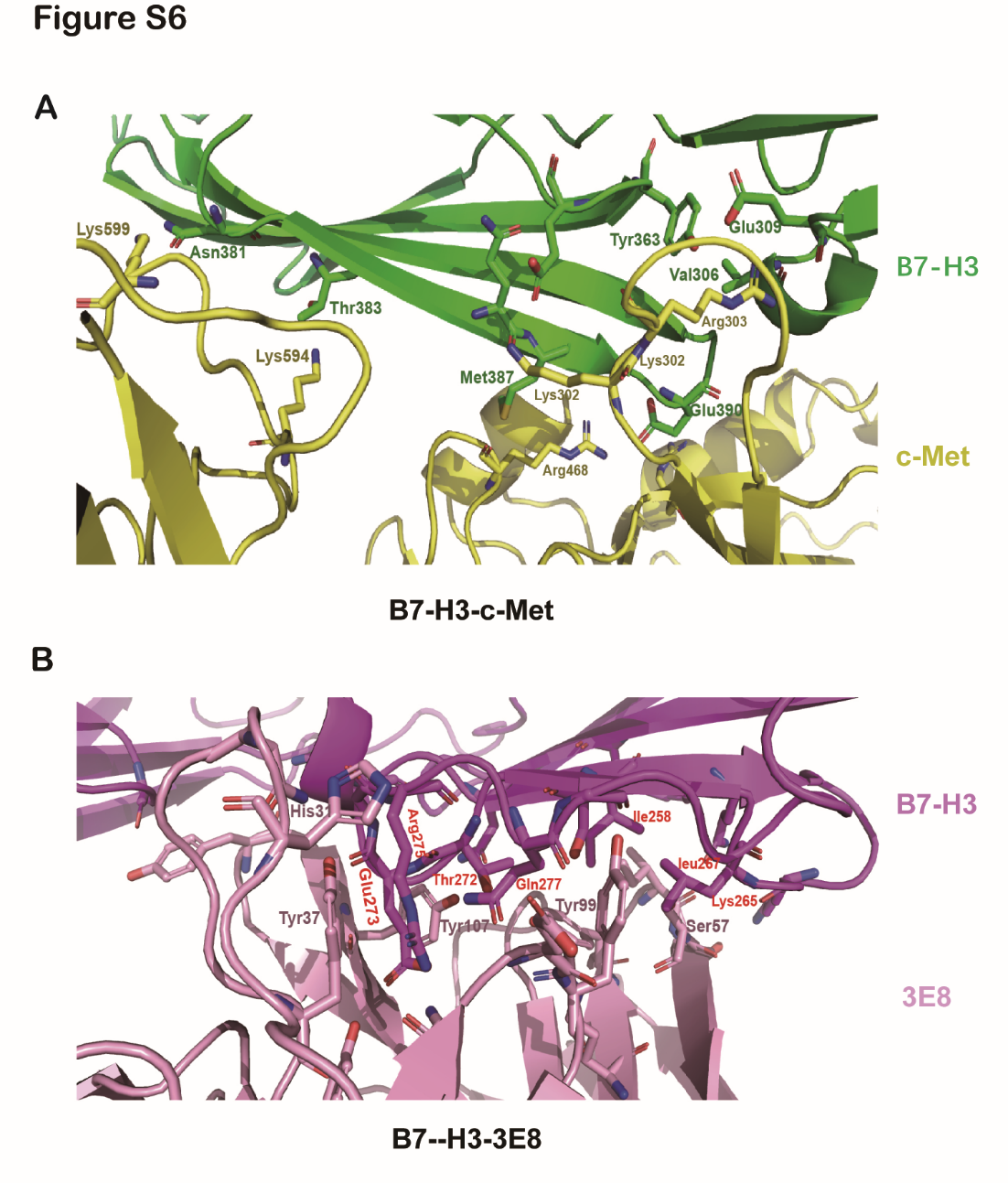


(A) Structural representation of the B7-H3/cMet complex interface, with key interacting residues depicted as sticks. B7-H3 is colored green, and cMet is shown in yellow.

(B) Critical interaction interface between B7-H3 and the 3E8 antibody, highlighting key residues (sticks). B7-H3 is rendered in magenta, while 3E8 is colored pink.

**Figure S7 Combination of B7-H3, c-Met and Stat3 provides better prognostic value for CRC and GBM tumor**


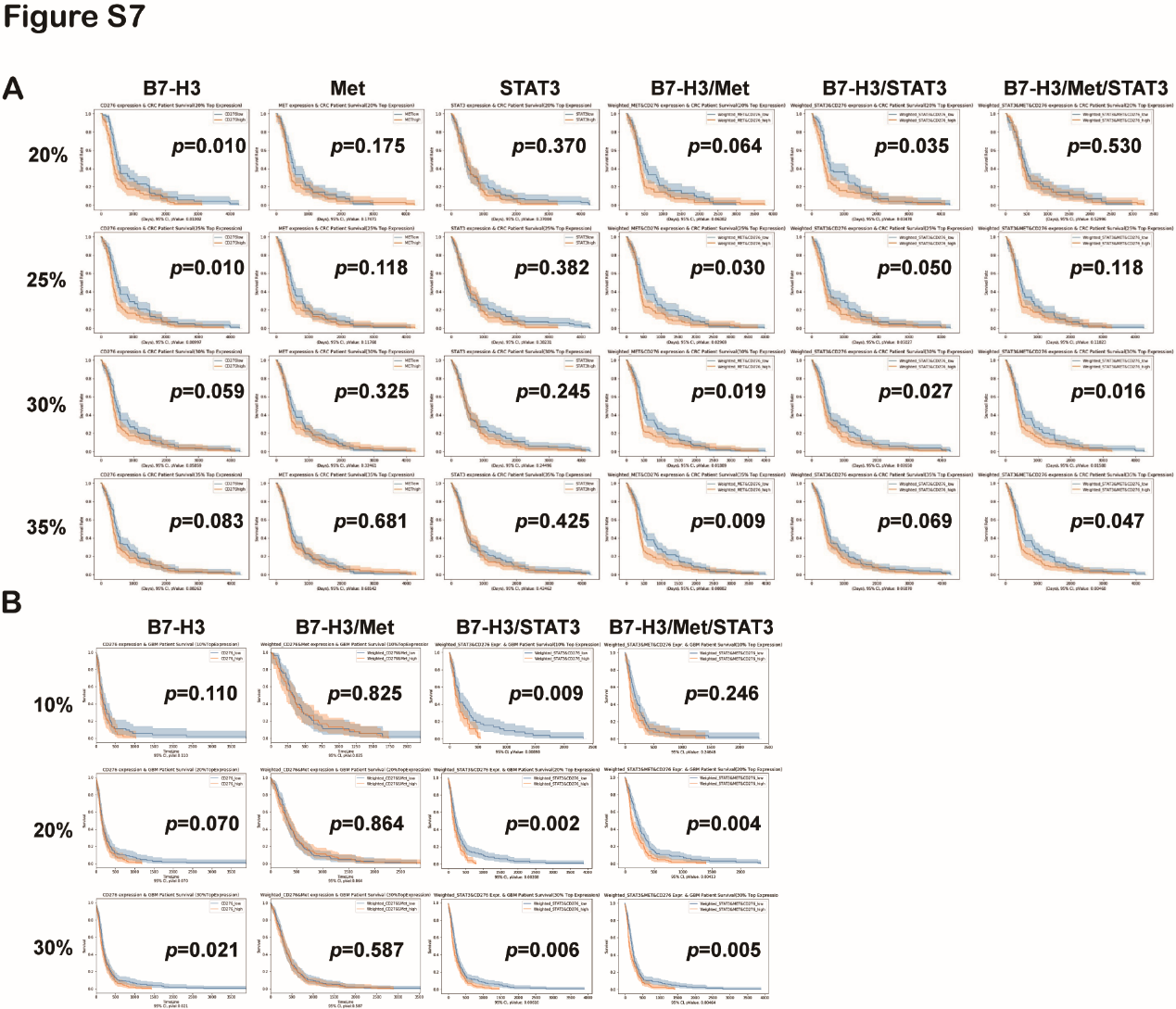


(A) Overall Survival information and gene expression data of CRC patients were extracted from the TCGA dataset. To describe the contribution of B7-H3/c-Met/STAT3 to patient survival time, Kaplan-Meier analysis was used here. We used the geometric mean of B7-H3/c-Met, B7-H3/STAT3, B7-H3/c-Met/STAT3 value to represent the weighted co-expression of B7-H3/c-Met, B7-H3/STAT3, B7-H3/c-Met/STAT3. In each dataset, we selected the 20, 25, 30, 35% of patients with either the highest or the lowest expression of target genes and compared the survival time. 95% CI and *P* value were calculated via log-rank test and shown at the bottom of each plot.

(B) Overall Survival information and gene expression data of GBM patients were extracted from the TCGA-CORD dataset. To describe the contribution of B7-H3/c-Met/STAT3 to patient survival time, we used Kaplan-Meier analysis. We used the geometric mean of B7-H3/c-Met, B7-H3/STAT3, B7-H3/c-Met/STAT3 value to represent the weighted co-expression of B7-H3/c-Met, B7-H3/STAT3, B7-H3/c-Met/STAT3. In each dataset, we selected the 10, 20, 30% of patients with either the highest or the lowest expression of target genes and compared the survival time. Similar trend is observed in GBM tumor. 95% CI and *P* value were calculated via log-rank test and shown at the bottom of each plot.

**Supplementary Table S1:** B7-H3 expression pattern in colorectal patients

|  |  | **B7-H3 expression^#^** | | | |
| --- | --- | --- | --- | --- | --- |
| **Characteristics** | **Total** | **Pattern**  **1** | **Pattern**  **2** | **Pattern 3** | ***P* value** |
| Gender |  |  |  |  | 0.029 |
| Male | 108 | 8 | 70 | 30 |  |
| Female | 89 | 17 | 54 | 18 |  |
| Age(year) |  |  |  |  | 0.263 |
| ＜65 | 125 | 18 | 79 | 28 |  |
| ≥65 | 72 | 7 | 45 | 20 |  |
| Tumor Volume |  |  |  |  | 0.825 |
| ＜5cm^3^ | 129 | 18 | 79 | 32 |  |
| ≥5cm^3^ | 68 | 7 | 45 | 16 |  |
| Duke's stage |  |  |  |  | 0.013 |
| A | 24 | 7 | 12 | 5 |  |
| B | 71 | 12 | 44 | 15 |  |
| C | 80 | 6 | 52 | 22 |  |
| D | 22 | 0 | 16 | 6 |  |
| LN metastases* |  |  |  |  | 0.004 |
| 0 | 104 | 19 | 65 | 20 |  |
| 1 | 47 | 3 | 33 | 11 |  |
| 2 | 42 | 3 | 24 | 15 |  |
| 3 | 4 | 0 | 2 | 2 |  |
| Distant metastases |  |  |  |  | 0.707 |
| No | 159 | 23 | 96 | 40 |  |
| Yes | 38 | 2 | 28 | 8 |  |
| Survival |  |  |  |  | 0.000 |
| Live | 85 | 20 | 52 | 13 |  |
| Dead | 112 | 5 | 72 | 35 |  |

# B7-H3 expression pattern: Pattern 1, no expression; Pattern 2, stromal cell expression; Pattern 3, tumor cell expression.

* 0-no lymph node metastases; 1-1~3 lymph nodes metastases; 2-4~6 lymph nodes metastases; 3-more than 7 lymph nodes metastases.

**Supplementary Table S2:** A panel of high score interacting molecules of B7-H3 were identified by mass spectrometry analyses


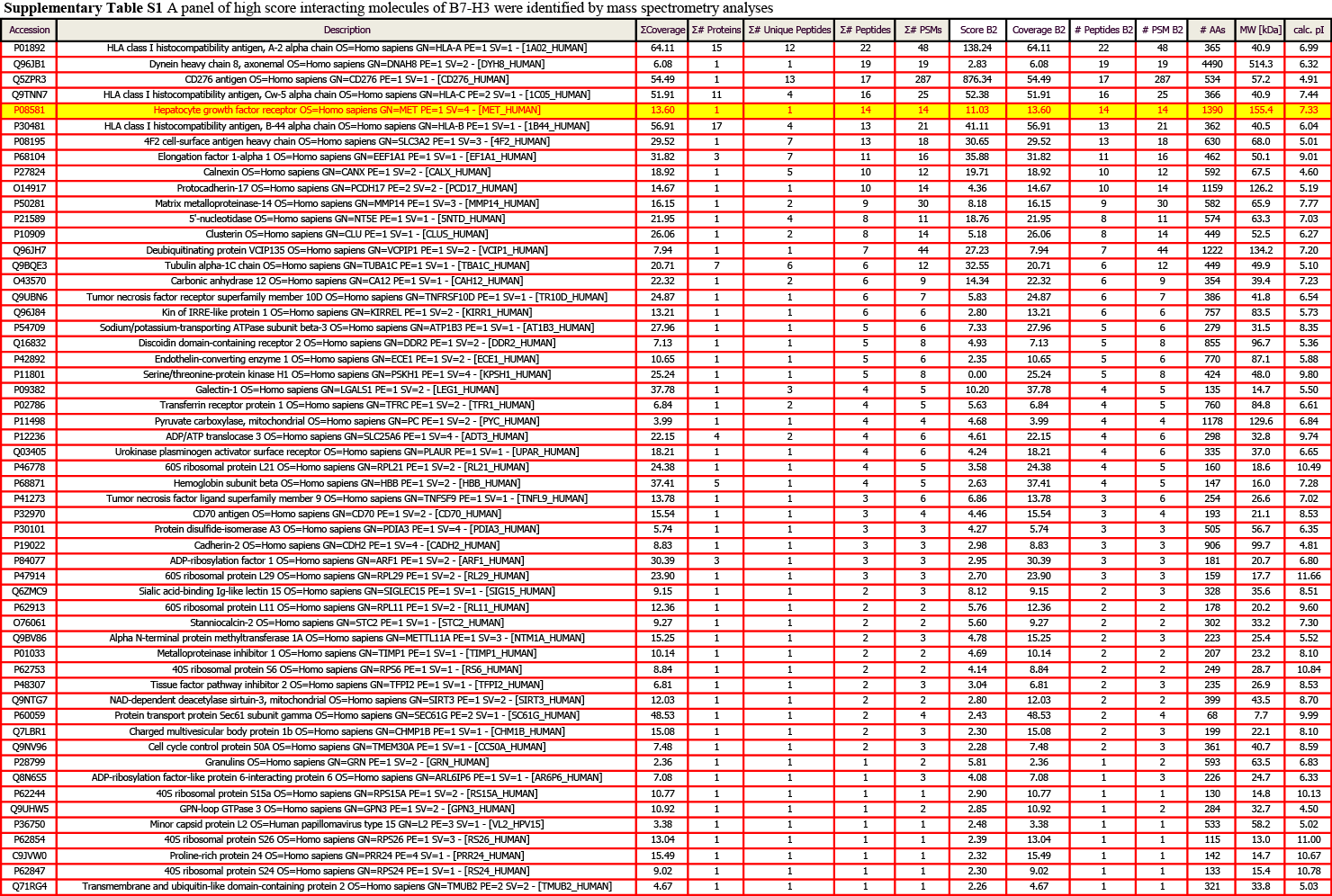


**Supplementary Table S3:** B7-H3 or c-Met expression pattern in colorectal patients

|  |  | | **B7-H3 expression^#^** | | | | | **c-Met expression** | | | |  | |  |
| --- | --- | --- | --- | --- | --- | --- | --- | --- | --- | --- | --- | --- | --- | --- |
| **Characteristics** | | **Total** | | **Pattern1** | | **Pattern 2** | ***P* value** | |  | **Low** | **High** | | ***P* value** | |
| Gender | |  | |  |  | | 0.221 | |  |  |  | | 0.101 | |
| Male | | 108 | | 78 | 30 | |  | |  | 63 | 45 | |  | |
| Female | | 89 | | 71 | 18 | |  | |  | 62 | 27 | |  | |
| Age(year) | |  | |  |  | | 0.400 | |  |  |  | | 0.923 | |
| ＜65 | | 125 | | 97 | 28 | |  | |  | 79 | 46 | |  | |
| ≥65 | | 72 | | 52 | 20 | |  | |  | 46 | 26 | |  | |
| Tumor Volume | |  | |  |  | | 0.844 | |  |  |  | | 0.792 | |
| ＜5cm^3^ | | 129 | | 97 | 32 | |  | |  | 81 | 48 | |  | |
| ≥5cm^3^ | | 68 | | 52 | 16 | |  | |  | 44 | 24 | |  | |
| Duke's stage | |  | |  |  | | 0.337 | |  |  |  | | 0.103 | |
| A | | 24 | | 19 | 5 | |  | |  | 17 | 7 | |  | |
| B | | 71 | | 56 | 15 | |  | |  | 48 | 23 | |  | |
| C | | 80 | | 58 | 22 | |  | |  | 49 | 31 | |  | |
| D | | 22 | | 16 | 6 | |  | |  | 11 | 11 | |  | |
| LN metastases* | |  | |  |  | | 0.028 | |  |  |  | | 0.226 | |
| 0 | | 104 | | 84 | 20 | |  | |  | 69 | 35 | |  | |
| 1 | | 47 | | 36 | 11 | |  | |  | 31 | 16 | |  | |
| 2 | | 42 | | 27 | 15 | |  | |  | 23 | 19 | |  | |
| 3 | | 4 | | 2 | 2 | |  | |  | 2 | 2 | |  | |
| Distant metastases | |  | |  |  | | 0.599 | |  |  |  | | 0.679 | |
| No | | 159 | | 119 | 40 | |  | |  | 102 | 57 | |  | |
| Yes | | 38 | | 30 | 8 | |  | |  | 23 | 15 | |  | |
| Survival | |  | |  |  | | 0.010 | |  |  |  | | 0.016 | |
| Live | | 85 | | 72 | 13 | |  | |  | 62 | 23 | |  | |
| Dead | | 112 | | 77 | 35 | |  | |  | 63 | 49 | |  | |

# B7-H3 expression pattern: Pattern 1, no expression or stromal cell expression; Pattern 2, tumor cell expression.

* 0-no lymph node metastases; 1-1~3 lymph nodes metastases; 2-4~6 lymph nodes metastases; 3-more than 7 lymph nodes metastases.

**Supplementary Table S4:** Sequences of siRNAs used in this study

| **Sequences** |
| --- |
| **siRNA sense(5’→3’) antisense（5’→3’）** |
| siNC UUCUCCGAACGUGUCACGUTT ACGUGACACGUUCGGAGAATT |
| si996 GCUGUCUGUCUGUCUCAUUTT AAUGAGACAGACAGACAGCTT |
| si1041 GUGCUGGAGAAAGAUCAAATT UUUGAUCUUUCUCCAGCACTT  siMet CCAGAGACAUGUAUGAUAATT UUAUCAUACAUGUCUCUGGTT |

**Supplementary Table S5:** Virus vector and target sequences used in this study

| **Lentiviral Vectors Specific Targeting Sequences** |
| --- |
| shNon pLVX-Puro TTCTCCGAACGTGTCACGT |
| shB7-H3 pLVX-Puro GUGCUGGAGAAAGAUCAAATT |

**Supplementary Table S6:** Real-time RT-PCR primers used in this study

| **Gene** | **Forward primer (5’- 3’)** | **Reverse primer (5’- 3’)** |
| --- | --- | --- |
| B7-H3 | ACAGGGCAGCCTATGACATT | CTGCATTCTCCTCCTCACAG |
| Actin | CATGTACGTTGCTATCCAGGC | CTCCTTAATGTCACGCACGAT |
| CD133 | AGTCGGAAACTGGCAGATAGC | GGTAGTGTTGTACTGGGCCAAT |
| c-Myc | GGCTCCTGGCAAAAGGTCA | CTGCGTAGTTGTGCTGATGT |
| Oct-4 | CTTGAATCCCGAATGGAAAGGG | GTGTATATCCCAGGGTGATCCTC |
| Nanog | TTTGTGGGCCTGAAGAAAACT | AGGGCTGTCCTGAATAAGCAG |
| Bmi1 | CCACCTGATGTGTGTGCTTTG | TTCAGTAGTGGTCTGGTCTTGT |
| Lgr5 | CTCCCAGGTCTGGTGTGTTG | GAGGTCTAGGTAGGAGGTGAAG |
| ABCB1 | TTGCTGCTTACATTCAGGTTTCA | AGCCTATCTCCTGTCGCATTA |
| ABCG2 | CAGGTGGAGGCAAATCTTCGT | ACCCTGTTAATCCGTTCGTTTT |
| c-Met | AGCAATGGGGAGTGTAAAGAGG | CCCAGTCTTGTACTCAGCAAC |
